# IL‐3 Modulates Microglia Polarization and Attenuates Neuroinflammation in Traumatic Brain Injury

**DOI:** 10.1002/advs.202504511

**Published:** 2026-03-31

**Authors:** Nana Huang, Qingchen Zhang, Yanrui Chen, Dapeng Yu, Ronghan Liu, Jianning Kang, Xiang Fang, Ying Zhang, Hong Bian, Yanxin Zhao, Yongcheng Yin, Ce Zhang, Yanfei Jia, Qingfa Chen, Yuepeng Fang, Shang Li, Fang Li, Zhengxin Jin, Bin Ning

**Affiliations:** ^1^ Central Hospital Affiliated to Shandong First Medical University Shandong First Medical University & Shandong Academy of Medical Sciences Jinan China; ^2^ Jinan Central Hospital Shandong University Jinan China; ^3^ School of Pharmacy Shandong University of Traditional Chinese Medicine Jinan China

**Keywords:** astrocytes, IL‐3, microglia, PRDX1, traumatic brain injury

## Abstract

Microglia play a crucial role in the progression of neuroinflammation following traumatic brain injury (TBI). Interleukin‐3 (IL‐3), a significant regulatory factor, has been involved in the pathogenesis of various diseases, yet its effects on neuroinflammation post‐TBI through microglia remain unclear. Here, we evaluate the potential of IL‐3 to alleviate neuroinflammation in microglia following TBI. Using the ABplex Multi‐Metric Streaming Joint Analysis to detect inflammatory factors, we observed significantly elevated levels of IL‐3 in cerebrospinal fluid, but not in blood samples, of patients with headaches and TBI. In addition, we found that administration of exogenous IL‐3 within the brain reduced neuroinflammation and promoted functional recovery in rat TBI models. Mechanistically, we identified Peroxiredoxin‐1 (PRDX1) as the target of IL‐3 in microglia. Notably, the protective effects of IL‐3 in TBI rats were abolished when PRDX1 was specifically knocked down in microglia. In conclusion, our experimental research demonstrates that IL‐3 acts as a key modulator via regulating microglia polarization to inhibit neuroinflammation. IL‐3 improves neurological function and prognosis in TBI rats by recruiting PRDX1 through IL‐3R to modulate microglia polarization. Therefore, IL‐3 may represent a novel therapeutic strategy for TBI.

## Graphical abstract

IL‐3 recruits PRDX1 through IL‐3R to modulate the inflammatory response of microglia via the KEAP1‐NRF2‐HO‐1/NF‐κB pathway to attenuate neuroinflammation in traumatic brain injury.

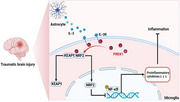

## 1 Introduction

Traumatic brain injury (TBI) is defined as damage to brain tissue caused by an external mechanical force to the head [1, 2]. Approximately half of the global population may experience one or more instances of T BIs over their lifetime, and around 50 million people suffer from TBIs annually, resulting in annual economic losses of over $400 billion [3, 4]. By 2030, TBI is projected to be one of the top three causes of trauma‐induced disability and death [4].

TBI is complex and multifaceted in pathophysiology, and is typically categorized into primary and secondary injuries [5]. Primary injury refers to the initial mechanical damage occurring at the moment of trauma. Following the mechanical injury [6], a series of cellular and biochemical changes, including inflammation, oxidative stress, mitochondrial dysfunction, and apoptosis, commence within minutes, leading to secondary injury. Evidence has accumulated that persistent and excessive secondary injury impedes neuroprotection and neurorepair, resulting in delayed and limited post‐TBI recovery of neurological function [7, 8]. Neuroinflammation is a major pathological process in this context [9]. Although preclinical studies have shown that anti‐inflammatory drugs can improve outcomes in animal models of TBI, results from randomized controlled clinical trials have been less promising [10]. Therefore, identifying new targets that may help restore the balance between pro‐inflammatory and anti‐inflammatory responses is essential for developing novel therapeutics and improving prognosis following TBI.

Microglia are the primary intrinsic immune cells of the central nervous system (CNS) and are considered the principal mediators of neuroinflammatory responses following CNS injury [11, 12, 13, 14, 15, 16]. Based on single‐cell sequencing and single‐cell mass cytometry, studies have identified various microglia states in both healthy and diseased brains. Microglia are no longer viewed as simply switching from “resting” to “activated” in response to injury, disease, or other challenges. Instead, microglia are continuously active, adopting different states, and responding to their surrounding environment to perform different functions in the context of health or disease. Modulating neuroinflammation is one of the most widely used therapeutic targets, with the core strategy being to intervene in microglia activation states through drugs or other means to reduce the release of harmful inflammatory factors and thereby mitigate neuronal damage [11].

Following brain trauma, resident microglia are rapidly activated and mobilized to the site of injury to clear debris and initiate the inflammatory cascade [17]. The activation of microglia is a crucial innate immune defense necessary for protecting the brain from further damage [18]. However, uncontrolled overactivation can be detrimental, potentially exacerbating neurodegenerative processes and causing neurological dysfunction [19]. Activated microglia exhibit functional polarization into two main phenotypes: (1) The pro‐inflammatory cytokines (TNF‐α, IL‐6) upregulate iNOS, and generate reactive oxygen species (ROS), leading to neurotoxicity and myelin damage [20]. (2) The anti‐inflammatory marker CD206 secretes mediators (IL‐10, Arg‐1, TGF‐β) that promote tissue repair and homeostasis [16, 21, 22]. Thus, regulating the inflammatory response of microglia can effectively reduce neuroinflammation caused by secondary injury [23, 24].

Interleukin‐3 (IL‐3) is a colony‐stimulating factor, with its downstream effects being mediated through its binding to the interleukin‐3 receptor (IL‐3R) [25]. IL‐3 influences transcriptional, morphological, and functional programming of microglia, providing them with an acute immune response program and enhanced motility [26]. IL‐3 regulates various inflammatory responses, which promote the rapid clearance of pathogens but also lead to pathological changes associated with chronic inflammation [27]. IL‐3 is associated with a range of diseases such as rheumatoid arthritis, multiple sclerosis, type 1 diabetes (T1D), asthma, and intestinal inflammation [27, 28, 29, 30, 31]. IL‐3 also mitigates collagen‐induced arthritis by regulating the development of Foxp3+ regulatory T cells [32]. IL‐3 exhibits unique roles in the CNS [33]. On one hand, in the brains of healthy mice and mice with Alzheimer's disease susceptibility genes, microglia increase IL‐3R expression and enhance their ability to clear Aβ and tau aggregates, thereby improving the cognitive function of the mice [26]. On the other hand, IL‐3‐IL‐3RA acts as a glial‐peripheral immune network that prompts immune cell recruitment to the CNS and worsens MS [29]. Previous studies have shown that IL‐3 and GM‐CSF can improve TBI by acting on bone marrow‐derived inflammatory cells (BINCs) [34]. While preliminary findings suggest a therapeutic potential for IL‐3 in TBI, the mechanisms remain unclear. It is unknown whether IL‐3 can modulate microglia polarization in TBI. Currently, research on TBI mainly focuses on the mechanisms of injury and pharmacological mechanisms, with the study of therapeutic targets for secondary brain injury being a focal point. Recent clinical evidence has demonstrated that therapeutic agents such as erythropoietin (EPO) and progesterone failed to exhibit neuroprotective efficacy [35, 36], highlighting the critical need for identifying novel targeted therapeutics as a research priority.

Peroxiredoxin‐1 (PRDX1), a member of the PRDX family, functions as a typical antioxidant enzyme and scavenges for ROS to inhibit oxidative stress‐induced cellular damage [37, 38, 39, 40, 41], thereby exerting protective antioxidant effects [42, 43]. PRDX1 is crucial for regulating the survival, inflammation, and oxidative stress of several resident cell types in the CNS, including microglia, astrocytes, oligodendrocytes, and neurons [44]. Nuclear factor erythroid‐2 related factor 2 (NRF2), one of the primary downstream effectors of PRDX1, is involved in various oxidative stress‐related molecular processes, including inflammatory responses, metabolic processes, and cell proliferation [45].

NRF2, a key regulator of endogenous defensive systems against oxidative stress, is generated by activated microglia stimulated by oxidative stress in the brain [46]. Previous research suggests that PRDX1 protects against pressure overload‐induced cardiac hypertrophy and heart failure by activating the NRF2/heme oxygenase‐1 (HO‐1) signaling pathway [47]. A deficiency in NRF2 is associated with enhanced inflammatory responses, whereas its up‐regulation can reduce pro‐inflammatory and immune responses regulated by NF‐κB transcription. NRF2 enhances antioxidant defenses and neutralizes ROS, thereby reducing ROS‐mediated NF‐κB activation [48]. The relationship between PRDX1 and TBI has not been previously reported; therefore, investigating the role of PRDX1 in TBI could potentially identify new targets for therapeutic interventions.

Here, we report that IL‐3 improves neurofunctional recovery and prognosis in TBI by enhancing the recruitment of PRDX1 via IL‐3R, which regulates microglial polarization and suppresses neuroinflammation. Our research shows that the administration of exogenous IL‐3 within the brain improved neurological function and prognosis in rat models of TBI. Mechanistically, IL‐3 enhances the recruitment of the IL‐3R to PRDX1, which modulates the Kelch‐like ECH‐associated protein 1 (KEAP1)‐NRF2‐HO‐1/NF‐κB pathway. We discover that IL‐3 is a crucial regulator of microglial polarization, potentially serving as a novel therapeutic strategy for TBI. These findings provide new insights into the pathogenesis of TBI and suggest new therapeutic strategies.

## 2 Results

### 2.1 IL‐3 is Predominantly Secreted by the CNS and May be Positively Correlated with Functional Recovery in Patients with TBI

**FIGURE 1:**
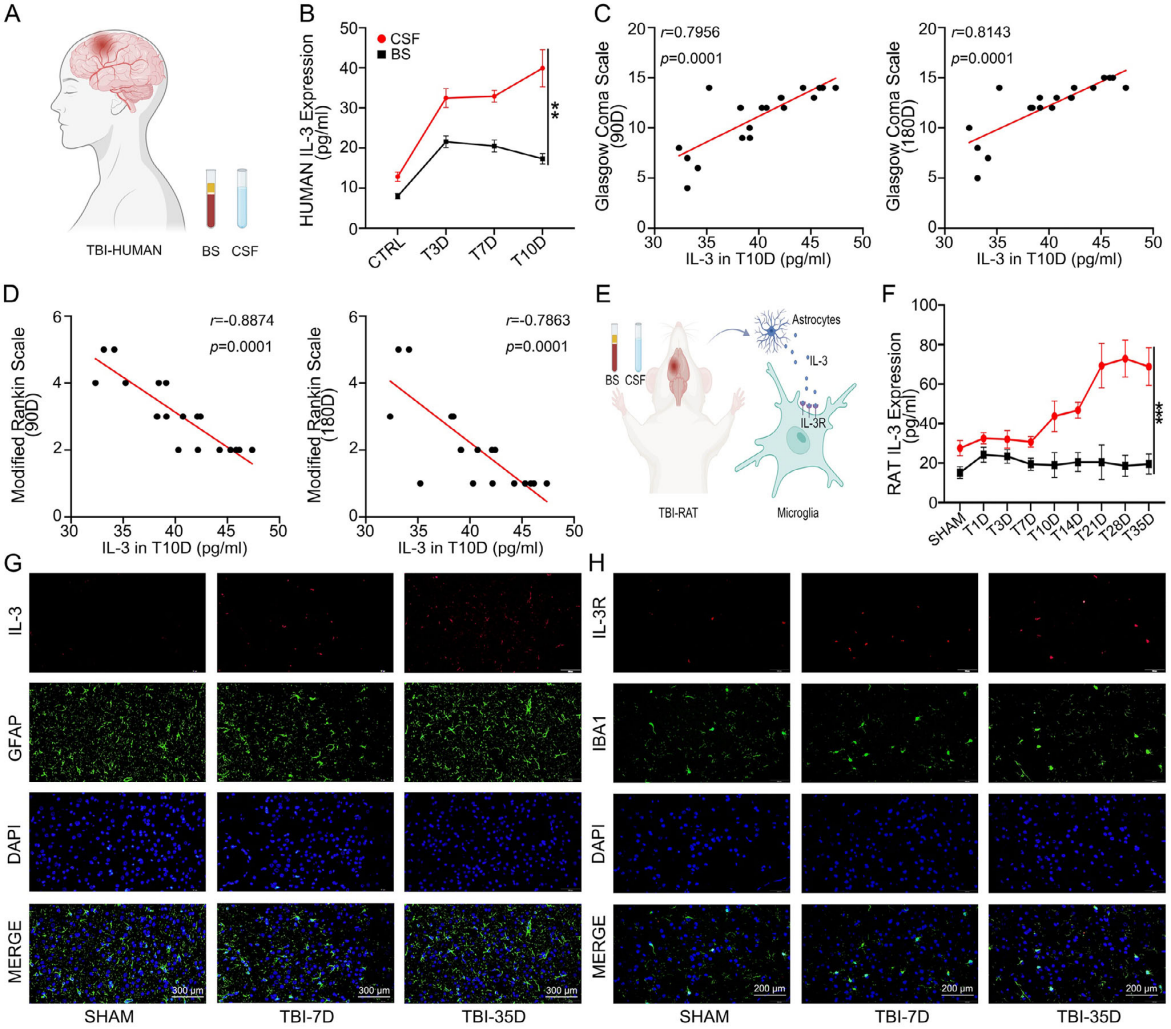
IL‐3 in CSF is significantly increased in TBI patients and may be positively correlated with functional recovery. A) Schematic diagram illustrating the extraction of CSF and blood serum (BS) from headache patients and TBI patients. *n* = 20 per group. B) Difference in the expression of IL‐3 in CSF and BS of TBI patients at different time points (day 3, day 7, and day 10 post‐injury) compared to headache patients. *n* = 20 per group. C) Correlation between IL‐3 expression on day 10 and the GCS at day 90 (*r* = 0.7956, *P* = 0.0001) and 180 days (*r* = 0.8143, *P* = 0.0001) in TBI patients. *n* = 20 per group. D) Correlation between IL‐3 expression on day 10 and the MRS at day 90 (*r* = ‐0.8874, *P* = 0.0001) and 180 days (*r* = ‐0.7863, *P* = 0.0001) in TBI patients. *n* = 20 per group. E) Schematic diagram illustrating the source and target cells of IL‐3 in rats with TBI. F) The expression level of IL‐3 in TBI rats at different time points (sham, day 1, day 3, day 7, day 10, day 14, day 21, day 28, and day 35). *n =* 11 per group. G) Representative IF images showing the expression levels of IL‐3 and GFAP in the TBI rat's brain at different time points. H) Representative IF images showing the expression levels of IL‐3R and IBA1 in TBI rats’ brains at different time points. Summary data are presented as the mean ± SD. ns, not statistically significant. ^*^ *P*<0.05, ^**^ *P*<0.01, ^***^ *P*<0.001, ^****^ *P*<0.0001. BS: Blood serum, CSF: Cerebrospinal fluid.

The CSF and BS samples from TBI patients (n = 20) were collected on days 3, 7, and 10, with corresponding samples from headache patients serving as negative controls (n = 20) (Figure 1A). The ABplex multi‐index flow analysis revealed that IL‐3 levels were consistently higher in CSF than in BS, with a significant increase observed on day 10 (Figure 1B). The expression levels of IL‐1β, IL‐4, IL‐6, and IL‐8 in CSF and BS peaked on day 3 and then decreased, showing no significant differences between CSF and BS (Figure S1A–D). The levels of IL‐10 and IL‐12 in CSF remained stable over time and were lower than those in BS (Figure S1E, F). TBI patients revealed significantly elevated IL‐3 levels in CSF at day 10 post‐injury. Correlation analysis revealed a positive correlation between IL‐3 levels in CSF on day 10 and Glasgow Coma Scale (GCS) on days 90 and 180 (*r* = 0.7956, *P* = 0.0001, *r* = 0.8143, *P* = 0.0001) (Figure 1C) and a negative correlation with the Modified Rankin Scale(MRS) on days 90 and 180 (*r* = ‐0.8874, *P* = 0.0001, *r* = ‐0.7863, *P* = 0.0001) (Figure 1D). A schematic diagram illustrates the source and target cells of IL‐3 in rats with TBI (Figure 1E). A similar trend was observed in IL‐3 concentrations in rats, suggesting that IL‐3 is predominantly generated in the brain (n = 11) (Figure 1F). IF analysis revealed IL‐3 co‐localization with astrocytic marker GFAP and showed significantly increased IL‐3 expression in late‐stage TBI rat brains. (Figure 1G). IF results from brain tissue sections of TBI rats at day 7 post‐injury indicate that IL‐3 showed no co‐localization with the neuronal marker NEUN, the microglia marker IBA1, or the oligodendrocyte marker MBP (Figure S2A–C). Schematic Diagram of astrocytes Induction and Collection (Figure S2D). In vitro experiments showed a significant increase in IL‐3 expression in astrocytes in response to TNF‐α (20 ng/ml) and IL‐1β (20 ng/ml) stimulation (Figure S2E). IF results from TBI rat brain tissue sections showed colocalization of the IL‐3R with IBA1, a microglia marker, indicating that IL‐3R is predominantly expressed in brain microglia (Figure 1H). These findings suggest that IL‐3 is secreted by astrocytes and acts on microglia in the brain, indicating that its high expression may be associated with functional recovery in TBI patients.

### 2.2 IL‐3 Improves Neurological Function and Prognosis in Rats with TBI by Reducing Inflammatory Responses

**FIGURE 2:**
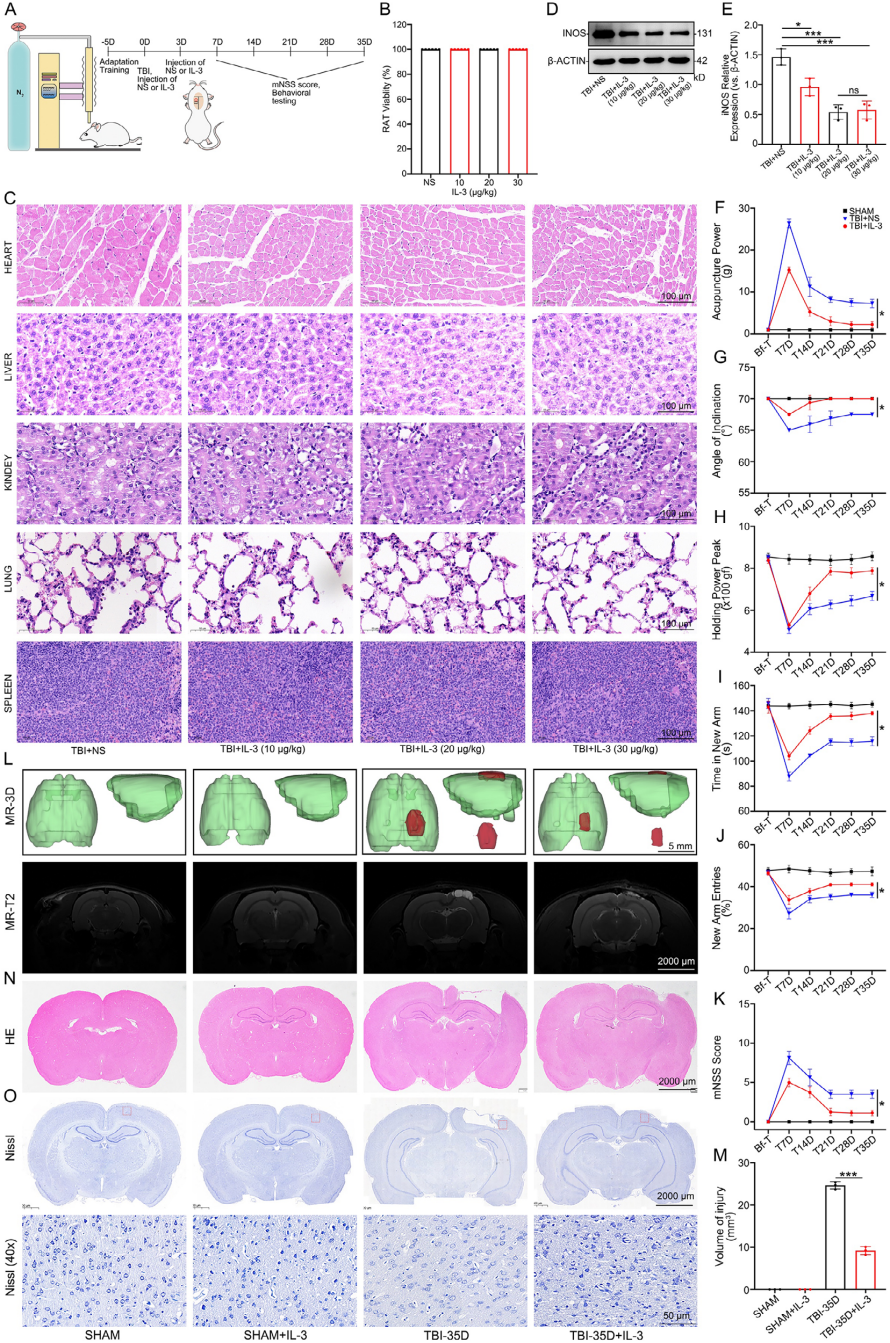
IL‐3 improves neurological function and prognosis in rats with TBI. A) Schematic diagram of the CCI model used to induce SD rats. B) The viability of healthy rats following injection with various concentrations of IL‐3. *n* = 12 per group. C) Histopathological evaluation using hematoxylin and eosin (H&E) staining was conducted to assess the toxicity of different concentrations of IL‐3 on the heart, liver, kidneys, lungs, and spleen in rats with TBI on day 35 post‐injury. *n* = 3 per group. D, E) Representative Western blot analysis demonstrated the expression levels of iNOS in the injured brain tissue of TBI rats on day 7 post‐injury, comparing the TBI+NS group with groups treated with IL‐3 at concentrations of 10 µg/kg, 20 µg/kg, and 30 µg/kg. *n* = 3 per group. F–K) Behavioral performance and mNSS of rats in the SHAM+NS group, TBI + NS group, and TBI + IL‐3 group at different time points (pre‐injury (Bf‐T) and day 7, day 14, day 21, day 28, and day 35 post‐injury). n = 8 per group. L–O) Performance of cranial MRI‐T2, HE, and Nissl staining at different time points (Bf‐T and day 35 post‐injury) in the SHAM group, SHAM+IL‐3 group, TBI‐35d group, and TBI‐35d+IL‐3 group. *n* = 3 per group. Summary data are presented as the mean ± SD. ns, not statistically significant. ^*^ *P*<0.05, ^**^ *P*<0.01, ^***^ *P*<0.001, ^****^ *P*<0.0001. TBI: Traumatic brain injury, TBI+NS: Traumatic brain injury treated with normal saline, TBI+IL‐3: Traumatic brain injury treated with Recombinant Rat IL‐3 (20 µg/kg) was injected using a brain stereotaxic apparatus on the immediate and 3rd day of injury. SHAM+IL‐3: SHAM treated with Recombinant Rat IL‐3 (20 µg/kg) was injected using a brain stereotaxic apparatus on the immediate and 3rd day of injury.

**FIGURE 3:**
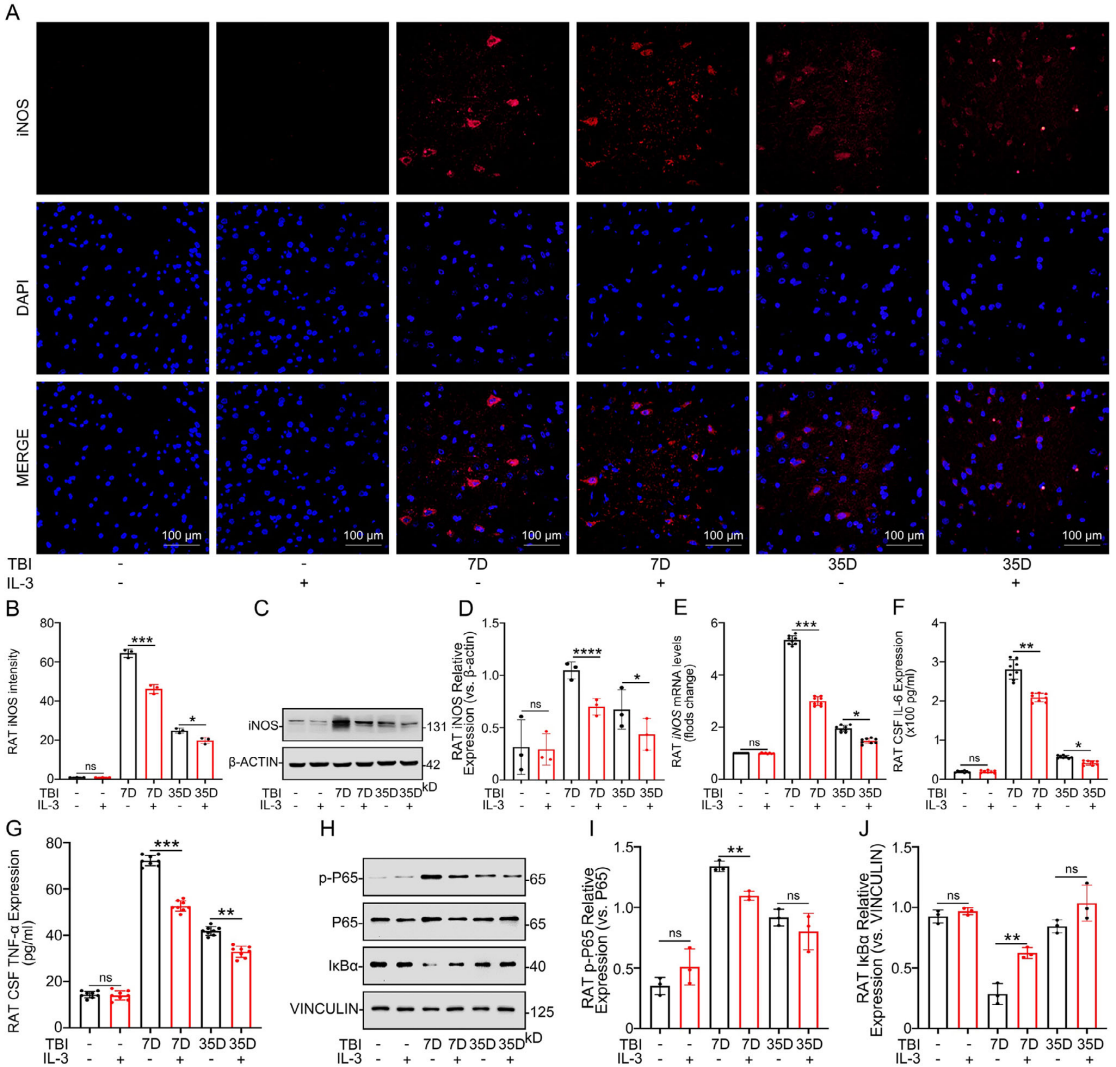
IL‐3 regulates the inflammatory responses in vivo. A, B) IF analysis showing iNOS expression levels in brain tissue in the TBI+NS group, and TBI +IL‐3 group at different time points (Bf‐T and day 7 and day 35 post‐injury). *n* = 3 per group. C, D) Representative WB analysis showing iNOS expression levels in brain tissue at the injury site in TBI rats with and without IL‐3 treatment. *n* = 3 per group. E) Quantification PCR of iNOS mRNA in the TBI+NS group and TBI +IL‐3 group at different time points (Bf‐T and day 7 and day 35 post‐injury), *n* = 8 per group. F, G) Expression levels of IL‐6 and TNF‐α in the TBI+NS group, and TBI +IL‐3 group at different time points (Bf‐T and day 7 and day 35 post‐injury), detected by ELISA. *n* = 8 per group. H–J) Representative Western blot analysis showing the expression levels of p‐P65 and IκBα in brain tissue at the injury site in the TBI+NS group and TBI +IL‐3 group at different time points (Bf‐T and day 7 and day 35 post‐injury). *n* = 3 per group. Summary data are presented as the mean ± SD. ns, not statistically significant. ^*^ *P*<0.05, ^**^ *P*<0.01, ^***^ *P*<0.001, ^****^ *P*<0.0001. TBI: Traumatic brain injury, TBI+NS: Traumatic brain injury treated with normal saline, TBI+IL‐3: Traumatic brain injury treated with Recombinant Rat IL‐3(20 µg/kg) was injected using a brain stereotaxic apparatus on the immediate and 3rd day of injury.

We used SD rats to directly evaluate the role of IL‐3 in TBI (n = 8). TBI was induced using a controlled cortical impact (CCI) model. Recombinant rat IL‐3 was injected into the left cortex of rats in the experimental group using brain stereotaxic methods on days 0 and 3 post‐injury, while NS injection was used in the negative control group (Figure 2A). We evaluated the safety and anti‐inflammatory effects of IL‐3 through systematic dose screening experiments (0, 10, 20, 30 µg/kg) in healthy rats. There were no significant differences in viability (Figure 2B). HE staining was performed to assess the extent of tissue damage in the heart, liver, kidneys, lungs, and spleen of TBI rats on day 35 post‐injury following IL‐3 administration at various concentrations (Figure 2C). All subjects were male Sprague‐Dawley rats. Body weight and food consumption remained comparable across cohorts. Western blot (WB) analysis of iNOS expression in TBI rats on day 7 post‐injury demonstrated that 20 and 30 µg/kg doses exhibited optimal anti‐inflammatory effects (Figure 2D,E). For in vivo experiments, an IL‐3 concentration of 20 µg/kg was selected for validation. Behavioral tests, including forelimb grip strength test, inclined plane test, mechanical allodynia test, the Y‐maze test, and the Modified Neurological Severity Score (mNSS) were conducted before‐TBI (Bf‐T) and on days 7, 14, 21, 28, and 35 post‐injury. The results showed that the TBI rats treated with IL‐3 exhibited significantly faster and better recovery compared to the TBI+NS group (Figure 2F–K). MRI‐T2 and HE staining revealed a significantly reduced injury range in IL‐3‐treated rats compared to the TBI group at day 35 post‐injury (Figure 2L–N). Nissl staining indicated that IL‐3 treatment led to enhanced neuronal repair in the brain injury area (Figure 2O). Additionally, qPCR, IF, and WB analyses showed a significant down‐regulation of iNOS, following IL‐3 treatment (Figure 3A–E). Similarly, ELISA results showed that the secretion of inflammatory factors such as IL‐6 and TNF‐α in the CSF of IL‐3‐treated rats was reduced (Figure 3F,G). On day 7 post‐TBI, WB results showed a significant down‐regulation of p‐P65 and up‐regulation of IκBα following IL‐3 treatment (Figure 3H–J). These results suggest that IL‐3 treatment effectively alleviates inflammatory responses to improve neurological function and prognosis in TBI rats by regulating the inflammatory response of microglia through the inhibition of iNOS, IL‐6, and TNF‐α expression.

### 2.3 IL‐3 Regulates the Inflammatory Response of Microglia and Possibly Acts by Inhibiting the NF‐κB Signaling Pathway In Vitro

**FIGURE 4:**
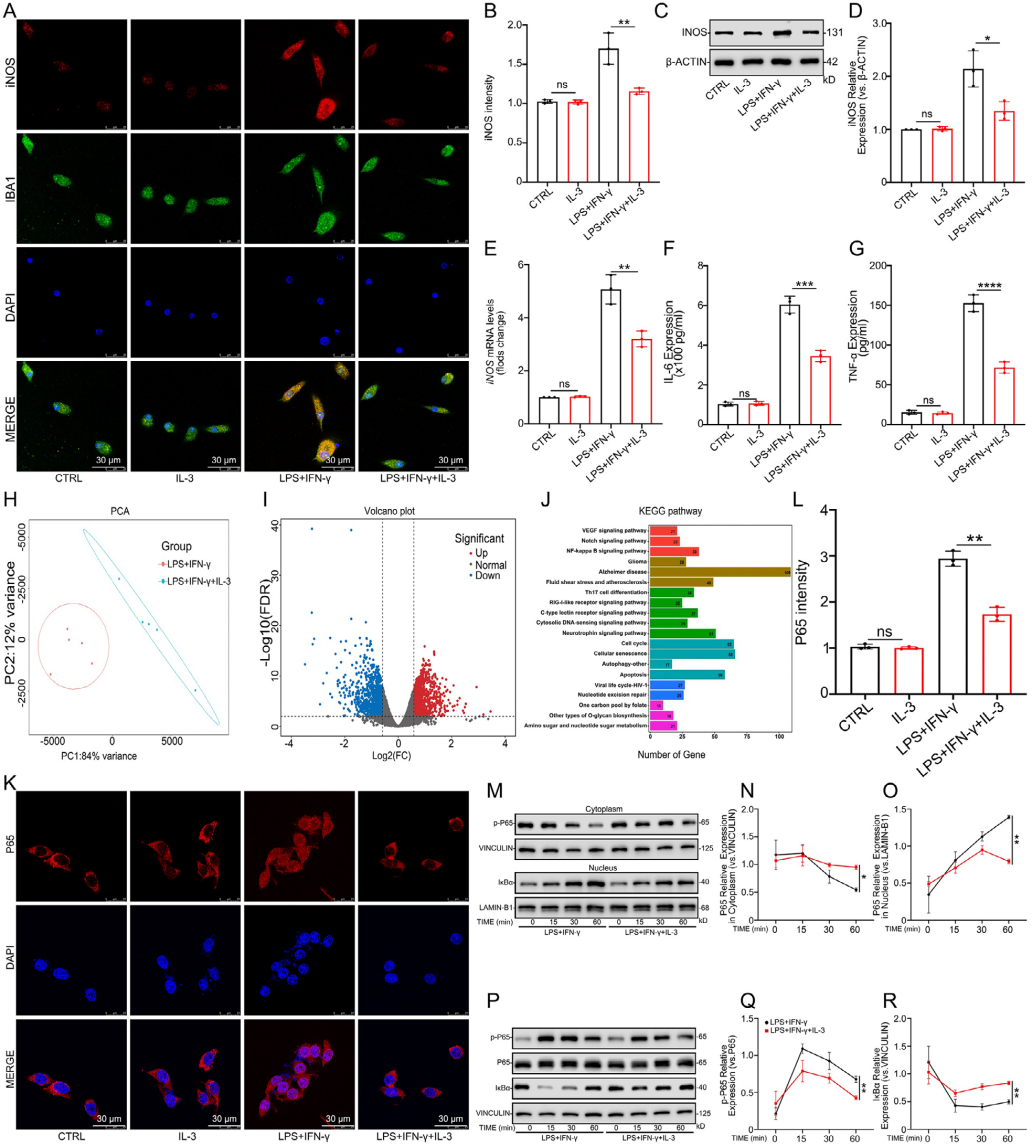
IL‐3 inhibited the inflammatory response of microglia via inhibiting the NF‐κB signaling pathway in vitro. A, B) Representative IF images showing the expression level of iNOS in primary microglia treated with or without IL‐3 before being exposed to LPS+ IFN‐γ. *n* = 3 per group. C, D) Representative WB analysis showing the expression levels of iNOS in primary microglia treated with or without IL‐3 before being exposed to LPS+IFN‐γ. *n* = 3 per group. E) Quantification of iNOS mRNA in primary microglia treated with or without IL‐3 before being exposed to LPS+IFN‐γ. *n* = 3 per group. F, G) The expression level of IL‐6 and TNF‐α in primary microglia treated with or without IL‐3 before treatment with LPS+IFN‐γ, detected by ELISA. *n* = 3 per group. H) PCA analysis of the LPS+IFN‐γ group and the LPS+IFN‐γ+IL‐3 group. I) Volcanic maps showing molecular differences among the LPS+IFN‐γ group, LPS+IFN‐γ+IL‐3 group. J) KEEG analysis of differentially expressed genes. K, L) Representative IF images showing the expression level of P65 in the microglia cytoplasm and nucleus with or without IL‐3 before being exposed to LPS+IFN‐γ. *n* = 3 per group. M–O) Representative WB analysis showing the expression levels of P65 in the microglia cytoplasm and nucleus with or without IL‐3 treatment at different time points (0, 15, 30, and 60 min). *n* = 3 per group. P–R) Representative WB analysis showing that the expression levels of p‐P65 and IκBα in microglia with or without IL‐3 treatment at different time points (0, 15, 30, and 60 min). *n* = 3 per group. Summary data are presented as the mean ± SD. ns, not statistically significant. ^*^ *P*<0.05, ^**^ *P*<0.01, ^***^ *P*<0.001, ^****^ *P*<0.0001. CTRL: Control, IL‐3: Recombinant mouse IL‐ 3 protein (Active) (40 ng/ml), LPS+IFN‐γ: LPS (100 ng/ml) and IFN‐γ (25 ng/ml). LPS+IFN‐γ+IL‐3: Recombinant mouse IL‐ 3 protein (Active) (40 ng/ml) was added 6 h earlier than LPS (100 ng/ml) and IFN‐γ (25 ng/ml).

Following IL‐3 pretreatment, IF, WB, and PCR analyses revealed down‐regulation of iNOS in primary microglia (Figure 4A–E). Following IL‐3 pretreatment, ELISA results showed reduced secretion of IL‐6 and TNF‐α in primary microglia (Figure 4F,G). These results indicate that IL‐3 inhibits microglia's inflammatory response in vitro. To understand the detailed mechanism by which IL‐3 affects microglia, RNA‐sequencing (RNA‐seq) was performed on microglia induced by LPS+IFN‐γ with or without IL‐3 pretreatment (n = 5 per group). PCA analyses of the LPS+IFN‐γ and LPS+IFN‐γ+IL‐3 groups showed distinct separation (Figure 4H). There were 2015 differentially expressed genes in the LPS+IFN‐γ group compared to the LPS+IFN‐γ+IL‐3 group, with 934 mRNAs significantly up‐regulated and 1081 mRNAs significantly down‐regulated (Figure 4I). The KEGG enrichment analysis revealed the involvement of major pathways, including NF‐κB, Notch, and VEGF signaling, with the NF‐κB pathway being significantly enriched (Figure 4J). IF experiments were performed to determine if IL‐3 could act on microglia via the NF‐κB signaling pathway. The results suggested that LPS+IFN‐γ stimulation promoted nuclear translocation of P65 in microglia, which was inhibited by IL‐3 pretreatment (Figure 4K,L). The WB results indicated that IL‐3 inhibited the nuclear transfer of P65 at different time points (0, 15, 30, and 60 min) (Figure 4M–O). Additionally, we assessed the expression of P65 and IκBα at different time points (0, 15, 30, and 60 min). The WB results showed that LPS+IFN‐γ promoted the phosphorylation of P65 and inhibited the expression of IκBα, while IL‐3 pretreatment interfered with these effects (Figure 4P–R). These findings indicate that IL‐3 may inhibit microglia's inflammatory response by suppressing iNOS, IL‐6, and TNF‐α expression through the NF‐κB pathway.

### 2.4 PRDX1 as a Downstream Target of IL‐3 in Microglia

**FIGURE 5:**
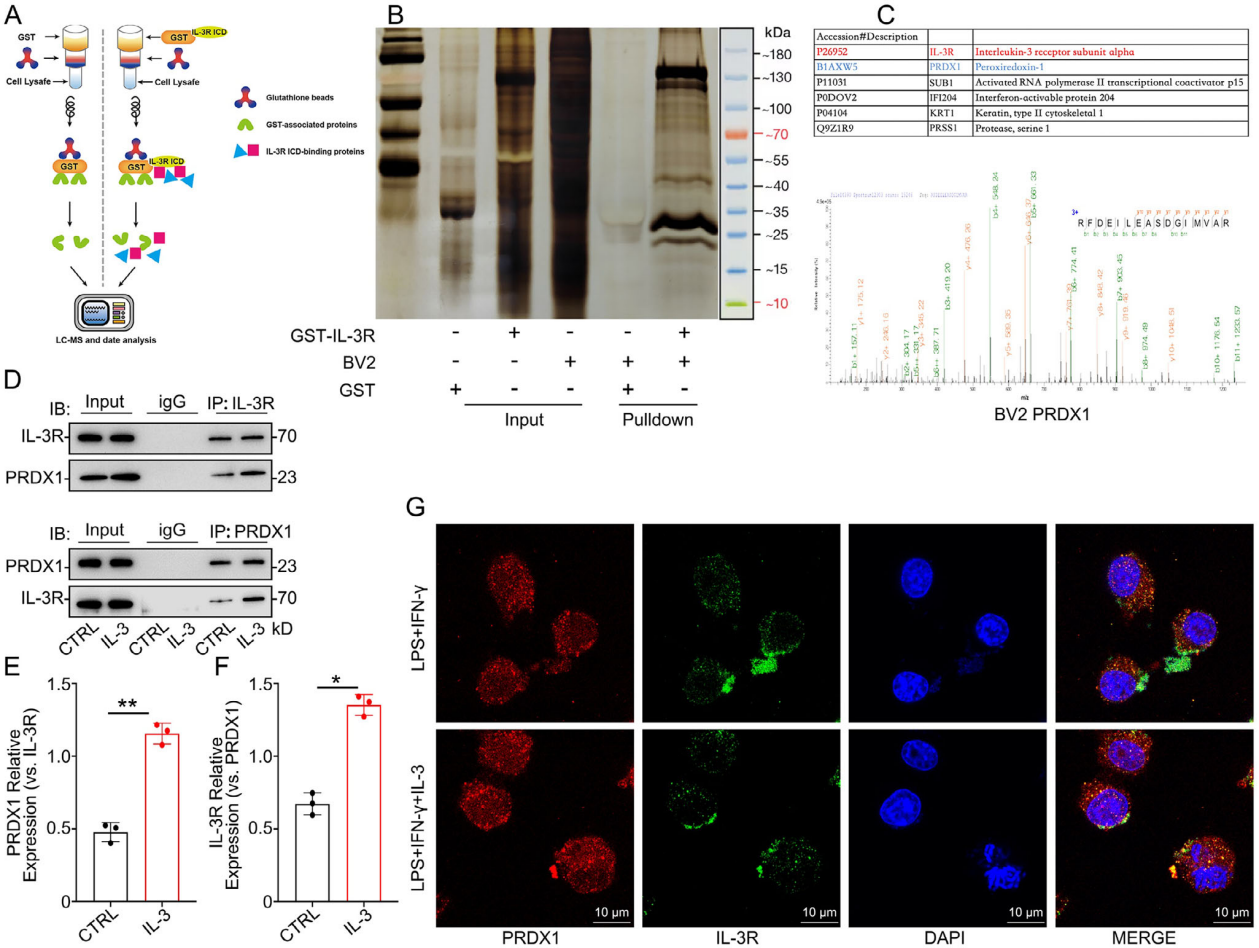
PRDX1 as a downstream target of IL‐3 in microglia. A) Schematic diagram of the GST‐Pulldown experiment. B) The Western blot of IL‐3R Pulldown. C) Mass spectrometry of PRDX1. D‐F) The Co‐IP analysis of IL‐3R‐PRDX1. G) Representative IF images showing the distribution of expression of PRDX1 and IL‐3R in the microglia treated with or without IL‐3 before being exposed to LPS and IFN‐γ. Summary data are presented as the mean ± SD. ns, not statistically significant. ^*^ *P*<0.05, ^**^ *P*<0.01, ^***^ *P*<0.001, ^****^ *P*<0.0001. CTRL: Control, IL‐3: Recombinant mouse IL‐3 protein (Active) (40 ng/ml), LPS+IFN‐γ: LPS (100 ng/ml) and IFN‐γ (25 ng/ml). LPS+IFN‐γ+IL‐3: Recombinant mouse IL‐ 3 protein (Active) (40 ng/ml) was added 6 h earlier than LPS (100 ng/ml) and IFN‐γ (25 ng/ml).

To identify the downstream targets of IL‐3 in microglia, we used GST‐pulldown assays to isolate proteins that are bound to IL‐3R following IL‐3 treatment (Figure 5A). The isolated proteins were analyzed using LC‐MS, revealing that PRDX1 is closely associated with inflammation in the nervous system (Figure 5B,C). Co‐IP assays confirmed that IL‐3 promotes the binding of IL‐3R to PRDX1 (Figure 5D–F). IF results further showed that IL‐3 promotes the translocation of PRDX1 to the cell membrane, promoting its association with IL‐3R (Figure 5G). These results suggest that PRDX1 may be a key target of IL‐3 in microglia.

### 2.5 IL‐3 Mitigates Neuroinflammation in TBI Rats via PRDX1

**FIGURE 6:**
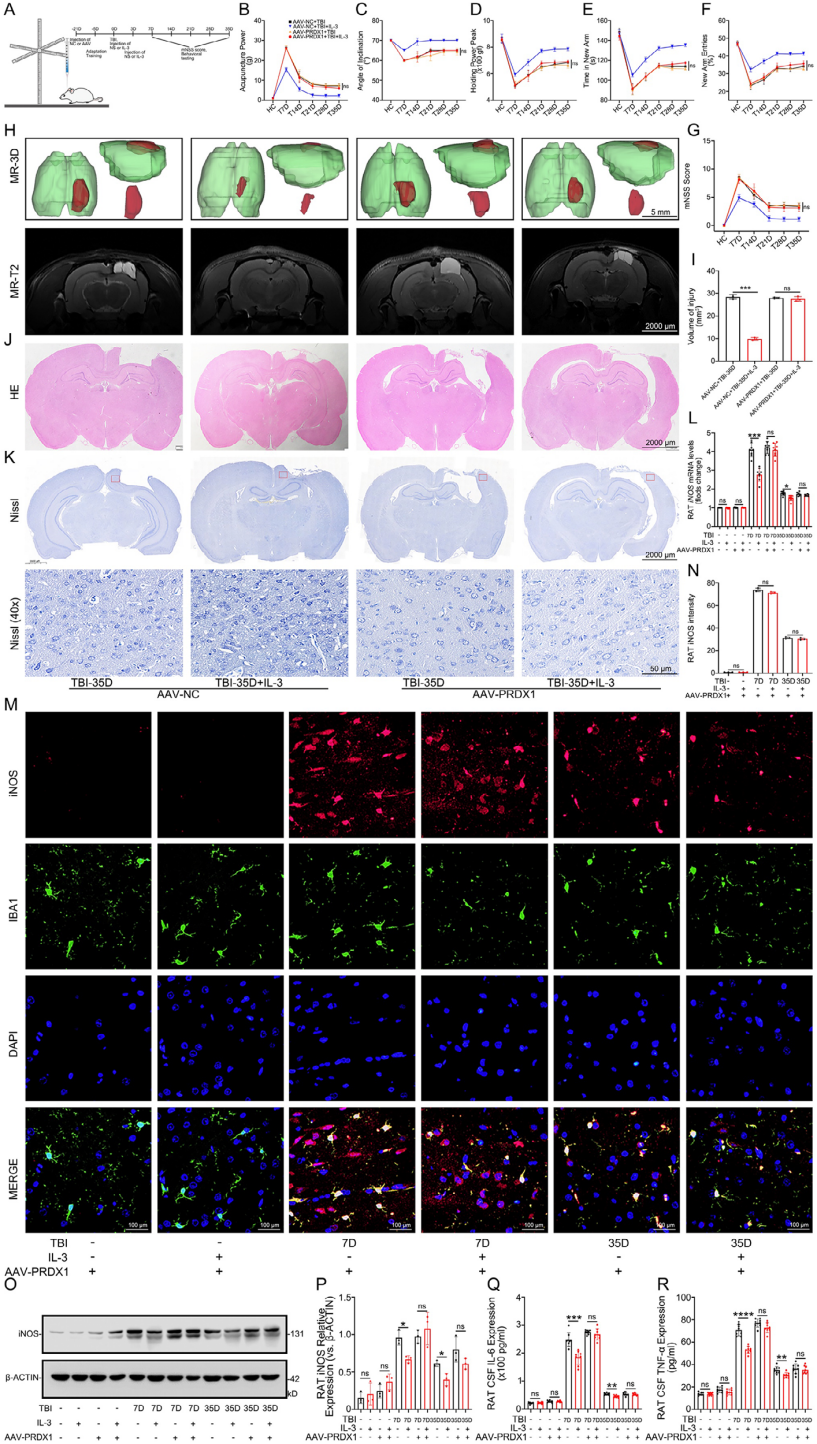
IL‐3 attenuates neuroinflammation following TBI via PRDX1. A) Schematic diagram of AAV‐Injection. B‐G) Behavioral performance and mNSS score of rats in AAV‐NC+TBI group, AAV‐NC+TBI+IL‐3 group, AAV‐PRDX1+TBI group, and AAV‐PRDX1+TBI+IL‐3 group at different time points (Bf‐T and day 7, day 14, day 21, day 28, and day 35 post‐injury). *n* = 8 per group. H‐K) Representative MRI‐T2, HE, and Nissi staining images of rat brain in AAV‐NC+TBI group, AAV‐NC+TBI+IL‐3 group, AAV‐PRDX1+TBI group, and AAV‐PRDX1+TBI+IL‐3 group at different time points (Bf‐T and day 35 post‐injury). *n* = 3 per group. L) The iNOS mRNA levels in the AAV‐NC+TBI group, AAV‐NC+TBI+IL‐3 group, AAV‐PRDX1+TBI group, and AAV‐PRDX1+TBI+IL‐3 group at different time points (Bf‐T and day 7, and day 35 post‐injury). *n* = 8 per group. M, N) Representative IF images showing the expression levels of iNOS in AAV‐PRDX1+TBI group, and AAV‐PRDX1+TBI+IL‐3 group at different time points (Bf‐T and day 7, and day 35 post‐injury). *n* = 3 per group. O, P) Representative WB analysis showing the expression levels of iNOS in AAV‐NC+TBI group, AAV‐NC+TBI+IL‐3 group, AAV‐PRDX1+TBI group, and AAV‐PRDX1+TBI+IL‐3 group at different time points (Bf‐T and day 7, and day 35 post‐injury). *n* = 3 per group. Q, R) The expression level of IL‐6 and TNF‐α in AAV‐NC+TBI group, AAV‐NC+TBI+IL‐3 group, AAV‐PRDX1+TBI group, and AAV‐PRDX1+TBI+IL‐3 group at different time points (Bf‐T and day 7, and day 35 post‐injury), detected by ELISA. *n* = 8 per group. Summary data are presented as the mean ± SD. ns, not statistically significant. ^*^ *P*<0.05, ^**^ *P*<0.01, ^***^ *P*<0.001, ^****^ *P*<0.0001. AAV‐NC+TBI: After transfecting with a negative control Adeno‐Associated Virus vector, a TBI model was established. AAV‐NC+TBI+IL‐3: After transfecting with a negative control Adeno‐Associated Virus vector, a TBI model was established, and treated with Recombinant Rat IL‐3 (20 µg/kg) was injected using a brain stereotaxic apparatus on the immediate and 3rd day of injury. AAV‐PRDX1+TBI: After transfecting with a PRDX1‐Knockdown Adeno‐Associated Virus vector, a TBI model was established. AAV‐PRDX1+TBI+IL‐3: After transfecting with a PRDX1‐Knockdown Adeno‐Associated Virus vector, a TBI model was established and treated with Recombinant Rat IL‐3 (20 µg/kg), which was injected using a brain stereotaxic apparatus on the immediate and 3rd day of injury.

To investigate the role of PRDX1 in the effects of IL‐3, PRDX1‐knockdown was performed by targeted injection of adenovirus AAV9‐IBA1 PRDX1 or control AAV vectors into the left cortex of rats using brain stereotaxic techniques (Figure 6A). Transfection efficiency was confirmed through GFP expression carried by the AAV after 21 days (Figure S3A). The WB analysis of brain tissue confirmed effective PRDX1‐knockdown (Figure S3B, C). Following PRDX1‐knockdown, recombinant rat IL‐3 was administered to the left cortex of rats with TBI using stereotaxic injection. IL‐3 treatment significantly improved performance in the forelimb grip strength test, inclined plane test, mechanical allodynia test, Y‐maze test, and mNSS in AAV‐NC; the reduction was not significant in rats with AAV‐PRDX1 (Figure 6B–G). Results from MRI‐T2 imaging and HE stains of post‐TBI rats revealed that while IL‐3 treatment reduced the injury area in rats injected with the AAV‐NC, the reduction was not significant in rats with AAV‐PRDX1 (Figure 6H–J). Nissl staining of brain tissue also supported these findings (Figure 6K). The qPCR, WB, and IF analyses of the injury site showed that AAV‐PRDX1 reduced the ability of IL‐3 to inhibit iNOS expression (Figure 6L–P). Similarly, IL‐3 inhibitory effects on inflammatory factors IL‐6 and TNF‐α were also compromised in the presence of AAV‐PRDX1 (Figure 6Q,R). These results suggest that IL‐3 may attenuate neuroinflammation and potentially enhance functional recovery in TBI rats, possibly through PRDX1‐mediated downre‐gulation of iNOS, IL‐6, and TNF‐α expression.

### 2.6 IL‐3 Regulates Microglia Inflammatory Response by PRDX1 to Modulate the NF‐κB Pathway

**FIGURE 7:**
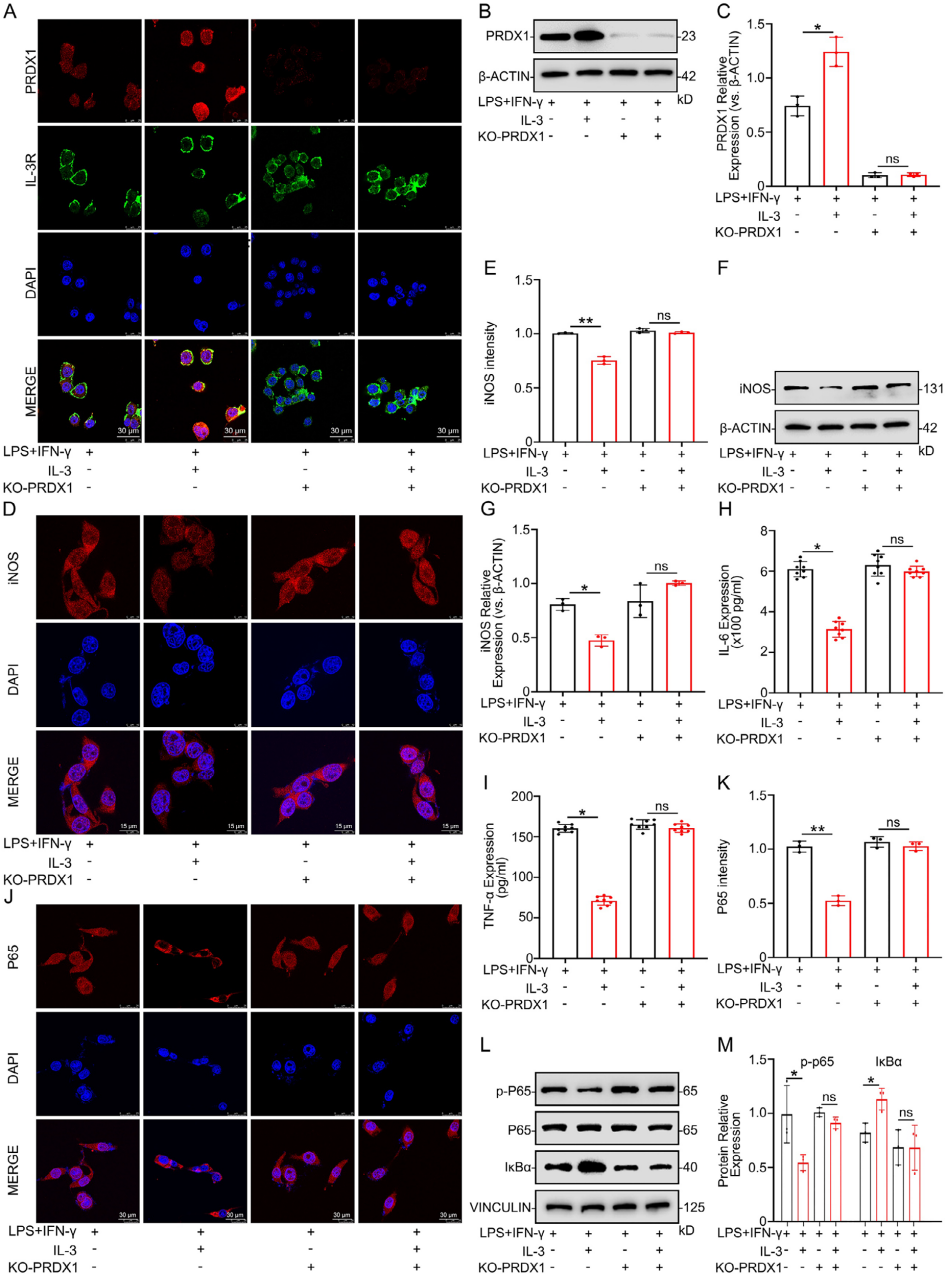
IL‐3 acts on PRDX1 to regulate the NF‐κB pathway and inhibit the inflammatory response of microglia. A) Representative IF images showing the distribution of PRDX1 and IL‐3R expression in microglia, with or without PRDX1‐knockout. *n* = 3 per group. B, C) Representative Western blot images showing the expression levels of PRDX1 in microglia treated with or without with or without IL‐3 after PRDX1‐knockout. *n* = 3 per group. D, E) Representative immunofluorescence showing the expression levels of iNOS in microglia treated with or without PRDX1‐knockout. *n* = 3 per group. F, G) Representative Western blot analysis showing the expression level of iNOS in microglia treated with or without PRDX1‐knockout. *n* = 3 per group. H, I) The expression level of IL‐6 and TNF‐α in microglia treated with or without PRDX1‐knockout, detected by ELISA. *n* = 8 per group. J, K) Representative IF images showing that the expression levels of P65 in microglia with or without PRDX1‐knockout. *n* = 3 per group. L, M) Representative Western blot images showing the expression levels of p‐P65 and IκBα in microglia with or without PRDX1‐knockout. *n* = 3 per group. Summary data are presented as the mean ± SD. ns, not statistically significant. ^*^ *P*<0.05, ^**^ *P*<0.01, ^***^ *P*<0.001, ^****^ *P*<0.0001. IL‐3: Recombinant mouse IL‐3 protein (Active) (40 ng/ml), LPS+IFN‐γ: LPS (100 ng/ml) and IFN‐γ (25 ng/ml). KO‐PRDX1: Transfection of lentivirus to knockout PRDX1 in microglia.

To further investigate the role of PRDX1 in IL‐3‐mediated microglia effects, microglia were transfected with sg‐PRDX1 (sgRNA) for PRDX1‐knockout. IF and WB analyses revealed a significant reduction in PRDX1 expression following sg‐RNA transfection (Figure 7A–C). Subsequent assessments revealed no significant differences in iNOS expression between IL‐3‐treated and untreated microglia in the PRDX1‐knockout condition (Figure 7D–G). ELISA results showed no significant differences in IL‐6 and TNF‐α levels between IL‐3 pretreated and non‐pretreated microglia under LPS+IFN‐γ stimulation in the PRDX1‐knockout condition (Figure 7H,I). Additionally, no significant differences were observed in P65 phosphorylation, IκBα expression, or nuclear translocation of P65 (Figure 7J–M). These results demonstrate that IL‐3 modulates microglial inflammatory response by suppressing iNOS, IL‐6, and TNF‐α expression through PRDX1‐mediated regulation of the NF‐κB pathway.

### 2.7 The KEAP1‐NRF2‐HO‐1 Signaling Pathway as a Key Mechanism for IL‐3 Regulation of Neuroinflammation Through PRDX1

**FIGURE 8:**
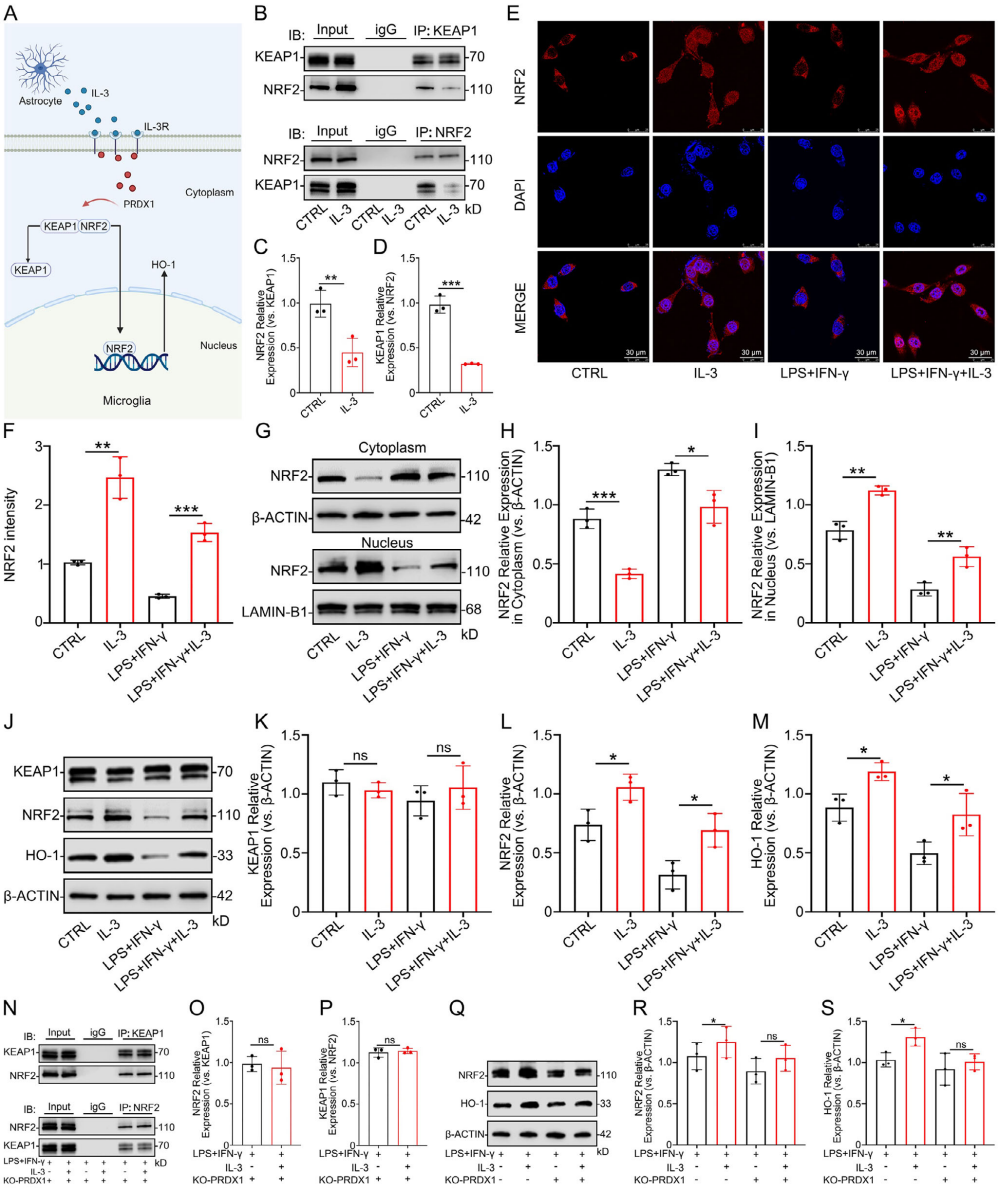
KEAP1‐NRF2‐HO‐1 signaling pathway mediates the effects of IL‐3 to regulate neuroinflammation via PRDX1. A) Diagram illustrating that IL‐3 modulates neuroinflammation by regulating the KEAP1‐NRF2‐HO‐1 signaling pathway through PRDX1. B‐D) Co‐IP analysis revealing the KEAP1‐NRF2 binding and dissociation in microglia. *n* = 3 per group. E, F) Representative IF images showing the expression levels of NRF2 in the microglia treated with or without IL‐3 before exposure to LPS+IFN‐γ. *n* = 3 per group. G–I) Representative WB images showing the expression levels of NRF2 in the cytoplasm and nucleus of microglia treated with or without IL‐3 before exposure to LPS+ IFN‐γ. *n* = 3 per group. J–M) Representative WB images showing the expression levels of KEAP1, NRF2, and HO‐1 in microglia treated with or without IL‐3 before being exposed to LPS+IFN‐γ. *n* = 3 per group. N‐P) Co‐IP analysis of the KEAP1‐NRF2 binding and dissociation in PRDX1‐knockout microglia. *n* = 3 per group. Q–S) Representative WB images showing the expression levels of NRF2 and HO‐1 in microglia treated with or without PRDX1‐knockout. *n* = 3 per group. Summary data are presented as the mean ± SD. ns, not statistically significant. ^*^ *P*<0.05, ^**^ *P*<0.01, ^***^ *P*<0.001, ^****^ *P*<0.0001. CTRL: Control, IL‐3: mouse IL‐ 3 protein (Active) (40 ng/ml), LPS+IFN‐γ: LPS (100 ng/ml) and IFN‐γ (25 ng/ml). LPS+IFN‐γ+IL‐3: Recombinant mouse IL‐3 protein (Active) (40 ng/ml) was added 6 h earlier than LPS (100 ng/ml) and IFN‐γ (25 ng/ml). KO‐PRDX1: Transfection of lentivirus to knockout PRDX1 in microglia.

Diagram illustrating that IL‐3 modulates neuroinflammation by regulating the KEAP1‐NRF2‐HO‐1 signaling pathway through PRDX1 (Figure 8A). To elucidate the involvement of the KEAP1‐NRF2‐HO‐1 signaling pathway in IL‐3′s effects on microglia, Co‐IP assays were conducted. Results showed that IL‐3 promoted the dissociation of KEAP1 from NRF2 in microglia (Figure 8B–D). IF and WB analyses showed that IL‐3 induced NRF2, which promoted its translocation into the nucleus (Figure 8E–I). The WB result revealed that IL‐3 treatment did not alter KEAP1 expression but significantly activated NRF2 and HO‐1 expression (Figure 8J–M). We further characterized the temporal expression profile of NRF2 in rat brain tissues following TBI. The WB result revealed that IL‐3 treatment significantly upregulated the expression of NRF2 (Figure S4A, B). In PRDX1‐knockout microglia, the Co‐IP analysis demonstrated that IL‐3 could not promote the dissociation of KEAP1‐NRF2, indicating that PRDX1 is crucial for this interaction (Figure 8N–P). The WB result showed no significant increase in NRF2 and HO‐1 levels with or without IL‐3 pretreatment in the context of LPS+IFN‐γ stimulation in PRDX1‐knockout microglia (Figure 8Q–S). These results confirm that IL‐3 inhibits iNOS, IL‐6, and TNF‐α to regulate microglia inflammatory response through PRDX1, potentially involving the KEAP1‐NRF2‐HO‐1 signaling pathway.

### 2.8 IL‐3 Modulates the NF‐κB Pathway via NRF2 to Suppress the Inflammatory Response of Microglia

**FIGURE 9:**
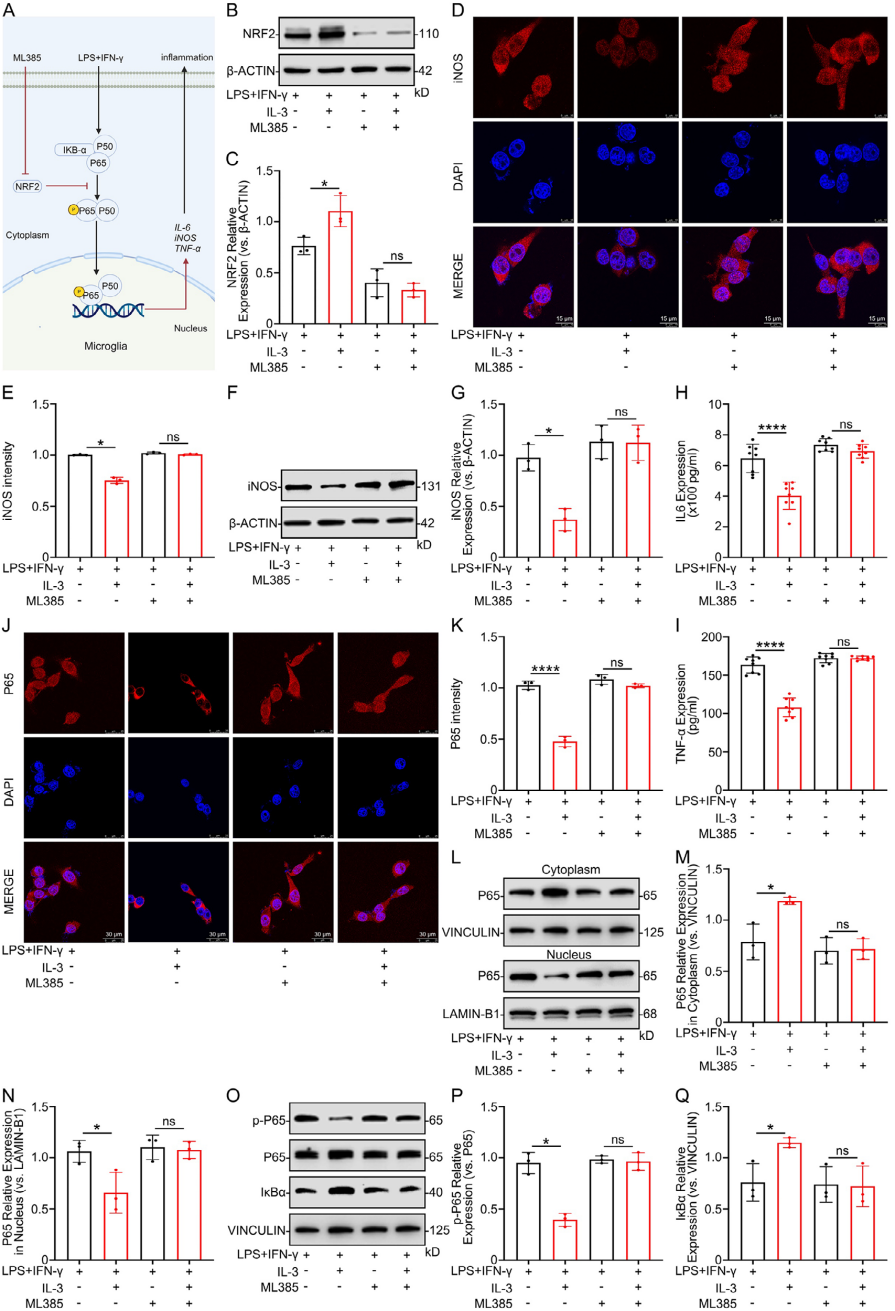
IL‐3 regulates the NF‐κB pathway through NRF2 to inhibit the inflammatory response of microglia. A) Diagram illustrating that IL‐3 inhibits the inflammatory response of microglia by regulating the NF‐κB pathway through NRF2. B, C) Representative WB images showing the expression levels of NRF2 in microglia treated with or without ML385. *n* = 3 per group. D, E) Representative IF images showing the expression levels of iNOS in microglia treated with or without ML385. *n* = 3 per group. F, G) Representative WB images showing the expression levels of iNOS in microglia treated with or without ML385. *n* = 3 per group. H, I) The expression level of IL‐6 and TNF‐α in microglia treated with or without ML385, detected by ELISA. *n* = 8 per group. J, K) Representative IF images showing the expression levels of P65 in the cytoplasm and nucleus of microglia treated with or without ML385. *n* = 3 per group. L–N) Representative WB images showing the expression levels of P65 in the cytoplasm and nucleus of microglia treated with or without ML385. O‐Q) Representative WB images showing the expression levels of p‐P65 and IκBα in microglia treated with or without ML385. Summary data are presented as the mean ± SD. ns, not statistically significant. ^*^ *P*<0.05, ^**^ *P*<0.01, ^***^ *P*<0.001, ^****^ *P*<0.0001. IL‐3: Recombinant mouse IL‐3 protein (Active) (40 ng/ml), LPS+IFN‐γ: LPS (100 ng/ml) and IFN‐γ (25 ng/ml). ML385: NRF2 knockdown in microglia.

Diagram illustrating that IL‐3 inhibits the inflammatory response of microglia by regulating the NF‐κB pathway through NRF2 (Figure 9A). To further test the hypothesis that the KEAP1‐NRF2‐HO‐1 pathway is involved in IL‐3′s modulation of microglia inflammation, NRF2 was inhibited using ML385, a specific NRF2 inhibitor. The results from WB confirmed effective inhibition of NRF2 (Figure 9B,C). From the IF and WB analyses, inhibition of NRF2 revealed that IL‐3 was unable to suppress iNOS expression (Figure 9D–G). Molecular level validation confirms that NRF2 is a key transcription factor in the IL‐3‐regulated NF‐κB pathway, affecting the release of inflammatory factors IL‐6 and TNF‐α (Figure 9H,I). ML385 counteracted IL‐3′s effect on the nuclear translocation of P65 (Figure 9J–N). Additionally, WB results showed that the ability of IL‐3 to inhibit P65 phosphorylation and promote IκBα expression was disrupted by NRF2 inhibition (Figure 9O–Q). These results suggest that IL‐3 inhibits the inflammatory response of microglia through PRDX1, potentially involving the KEAP1‐NRF2‐HO‐1 signaling pathway.

## 3 Discussion

Our study reveals that IL‐3 is primarily secreted by the CNS and is positively correlated with neurological function and prognosis in TBI rats. We demonstrate that IL‐3 promotes the recruitment of PRDX1 to the IL‐3R, thereby inhibiting the inflammatory response of microglia. This process significantly improves neurological function and prognosis in rats with TBI. Additionally, our findings show that PRDX1 is essential in the IL‐3 regulation of the KEAP1‐NRF2‐HO‐1/NF‐κB pathway. These findings indicate that IL‐3 exerts a protective effect in TBI rats by regulating microglia polarization, thus offering a novel therapeutic strategy for TBI treatment.

Recent research has focused on inhibiting the neuroinflammatory response following TBI to promote neuron recovery and axon regeneration [49, 50, 51, 52, 53]. Addressing neuroinflammation post‐TBI to improve neurological function and prognosis remains an urgent, critical clinical challenge [54, 55]. Analysis of clinical specimens from TBI patients revealed that IL‐3 levels in cerebrospinal fluid were significantly higher than in serum. Moreover, high IL‐3 expression in the mid‐to‐late stages of TBI was positively correlated with neurological function and prognosis, indicating a protective role for IL‐3 in TBI.

TBI triggers rapid activation of microglia, leading to the release of large amount of pro‐inflammatory and cytotoxic mediators and triggering a cascading inflammatory response. As the primary mediators of innate immune responses in the CNS, microglia play a crucial role in neuroinflammation [24, 54, 56]. Previous studies have shown that IL‐3 is released by astrocytes in the brain and acts on microglia, and can improve neuropathology and cognitive function in Alzheimer's disease (AD) [26]. Recent research has highlighted the therapeutic potential of IL‐3 in various diseases. For instance, Karen et al. demonstrated the beneficial role of IL‐3 in chronic colitis by promoting T cell mucosal residency through cytoskeletal changes [57]. Rupesh et al. found that IL‐3 strongly inhibits osteoclast formation, pathological bone resorption, and inflammatory arthritis, thus reducing the production of pro‐inflammatory cytokines IL‐6, IL‐17A, TNF‐α, and IL‐1, while increasing the production of the anti‐inflammatory cytokine IL‐10 in collagen‐induced arthritis mice [32]. However, the specific effects of IL‐3 on neuroinflammation after TBI via microglia remained unclear. Our study revealed a significant upregulation of IL‐3 secretion in cerebrospinal fluid during the intermediate‐to‐late phases of TBI in rat models. Early therapeutic intervention effectively attenuates acute neuroinflammation, minimizes secondary damage, enhances neurogenesis and synaptic reorganization, while reducing oxidative stress. In contrast, delayed treatment coincides with established chronic inflammation, compromised neural regenerative capacity, and closure of the critical recovery window. These pathophysiological considerations dictate the necessity for early intervention [58]. Our experimental findings further demonstrate that IL‐3 administration in the early stage of TBI attenuates neuroinflammation and improves functional outcomes. Our study further explores the role and specific mechanisms of IL‐3 in regulating microglia polarization in TBI. We found that upregulation of IL‐3 suppresses the expression of inflammatory factors such as iNOS, IL‐6, and TNF‐α, and modulates he inflammatory response of microglia, thereby mitigating neuroinflammation and improving neurological function and prognosis in TBI rats. Our research findings demonstrate the protective effects of IL‐3 on TBI rats through microglia polarization regulation, potentially paving the way for new therapeutic approaches for TBI patients.

We identified PRDX1 as a novel target of IL‐3 acting through IL‐3R on microglia. However, it remains unclear whether the protective effect of IL‐3 after TBI is dependent on the presence of PRDX1 in microglia. PRDX1 plays a central role in maintaining cellular redox balance and responding to redox imbalances [47], and it is also involved in regulating inflammatory responses [59]. Sinai et al. found that PRDX1 exerts strong antioxidant effects in cancer therapy [60]. Furthermore, studies have shown that PRDX1 can prevent brain injury by reducing oxidative stress [61, 62, 63]. Our research confirms that PRDX1 is critical for IL‐3‐regulated microglia polarization. When PRDX1 is ablated in microglia within the brains of TBI rats, the protective effects of IL‐3 on the CNS are significantly diminished. This suggests that IL‐3 promotes the recruitment of PRDX1 through IL‐3R, thereby modulating the inflammatory response of microglia to alleviate neuroinflammation and improve neurological function and prognosis in TBI rats.

The correlation between IL‐3 and NRF2 has not been previously reported. PRDX1 serves as a crucial scavenger of ROS, while the KEAP1/NRF2 pathway represents the primary cellular defense mechanism against oxidative stress. Notably, PRDX1 overexpression can elevate NRF2 levels and its downstream antioxidant proteins [47]. NRF2 confers protection against neurological diseases by combating oxidative stress, inhibiting inflammation, and reducing cell death [51, 64, 65, 66]. Keap1‐NRF2, a key regulator of endogenous defensive systems against oxidative stress, is produced by active microglia induced by oxidative stress in the brain [67, 68]. Activated NRF2 translocases to the nucleus to initiate the transcription of various genes, particularly HO‐1 [69]. Positive regulation of NRF2 activity post‐TBI can reduce oxidative stress and post‐traumatic inflammation, thereby improving clinical outcomes [70]. NRF2 also prevents the degradation of IκBα, thereby blocking the nuclear translocation of NF‐κB and the transcription of pro‐inflammatory genes [46, 71]. Our results demonstrate for the first time that IL‐3 promotes the recruitment of PRDX1 by the IL‐3R, facilitates the dissociation of KEAP1‐NRF2, enables NRF2 to enter the nucleus, and enhances HO‐1 transcription. This further inhibits the NF‐κB pathway, thereby inhibiting the inflammatory response of microglia and reducing neuroinflammation.

This study has limitations. First, the sample size of TBI patients was relatively small, necessitating an expansion of the sample size in future large‐scale studies. Second, the underlying mechanisms of IL‐3 action require further exploration. Thirdly, MRI infarct volume measurements conducted during the subacute phase (particularly at 7 and 35 days) may indeed be influenced by incomplete resolution of vasogenic edema or signal artifacts caused by blood breakdown products. These factors could lead to over‐ or underestimation of the true infarct core size. In this study, we acknowledge and recognize that these potential confounding factors affect lesion segmentation and volume calculations for all subjects and all groups at the specified time points (7 and 35 days). This represents one of the inherent limitations of using MRI during this period for infarct volume assessment. The therapeutic application of IL‐3 in the treatment of TBI requires additional clinical trials to validate its effectiveness.

## 4 Conclusion

In summary, this study reveals the crucial role of IL‐3 in regulating microglia polarization. We report that IL‐3 regulates microglia polarization to improve neurological function and prognosis in rats with TBI by recruiting PRDX1 through the IL‐3R. Our findings confirm the critical role of PRDX1 in the IL‐3‐mediated regulation of the KEAP1‐NRF2‐HO‐1/NF‐κB pathway. This research provides novel insights into TBI treatment, deepening the understanding of TBI mechanisms and offering new perspectives for the clinical management of traumatic brain injuries. These results may lead to the development of new therapeutic strategies for the repair and regeneration of neurological function following TBI.

## 5 Experimental Section

### 5.1 Human Samples

Twenty patients with severe TBI and 20 age‐matched headache patients were recruited from the Affiliated Central Hospital of Shandong First Medical University in Jinan, Shandong, China. The inclusion criteria were: (1) meeting clinical diagnostic criteria for TBI, with GCS [72] score of 3–8, (2) aged 18–60 years old, (3) admitted into the hospital within 24 h of trauma. The exclusion criteria were: (1) complicated with a brain tumor, (2) organic disease of the heart, lung, liver, kidney, etc., (3) disease of the blood system and coagulation dysfunction, (4) pregnant women. Twenty patients with headache who were admitted to the Affiliated Central Hospital of Shandong First Medical University were selected as the control group, and the inclusion criteria were: (1) aged 18–60 years old (2) admitted to our hospital. The exclusion criteria for controls were: (1) inflammatory diseases of the central nervous system, (2) infectious and neoplastic diseases, (3) cerebrovascular diseases, (4) abnormal cerebrospinal fluid routine, protein glucose chloride, DNA determination of various pathogens, etc., (5) blood system diseases and coagulation dysfunction, (6) organic diseases of the heart, lung, liver, kidney, etc., (7) pregnant women. Blood serum (BS) and cerebrospinal fluid (CSF) were collected from TBI patients on day 3, day 7, and day 10, respectively. Serum and cerebrospinal fluid (CSF) samples were collected from headache patients upon hospital admission. The severity of TBI was assessed using the Glasgow Coma Scale (GCS) on post‐injury days 1, 3, 10, 90, and 180. We defined mild TBI as the Glasgow Coma Scale (GCS) score of 13–15, moderate TBI as the GCS score of 9–12, and severe TBI as the GCS score of 3–8. TBI severity was assessed using the modified Rankin Scale (MRS) at 90‐ and 180‐days post‐injury [73]. The severity of TBI was categorized according to MRS core as follows: 0‐asymptomatic, 1‐symptomatic but no apparent disability, 2‐mild disability, 3‐moderate disability, 4‐moderately severe disability, 5‐severe disability, and 6‐death. CSF was obtained by lumbar puncture. Both CSF and BS were centrifuged at 3000 g for about 10 min at 4 ℃ to remove cells and debris, and the supernatant was carefully decanted into a new RNase‐free centrifuge tube (be careful not to transfer the precipitate) and centrifuged at 13 000 g for about 2 min at 4 ℃ to remove the residual debris and cells. The supernatant was transferred to a new −192 ℃ ultra‐low temperature threaded freezing tube, frozen in liquid nitrogen for 1 h, sealed tightly, and stored at −80 ℃. The above steps were performed within 1 h after the sample collection.

The study protocol was approved by the Medical Ethics Committee of the Central Hospital of Shandong First Medical University in Jinan on July 15, 2022 (approval number: 2022‐077‐02). Written informed consent was obtained from all participants. Research has been conducted in accordance with the ethical principles for medical research involving human subjects expressed in the Declaration of Helsinki.

### 5.2 ABplex Multi‐Metric Streaming Joint Analysis

Human BS and CSF were collected to test for IL‐1β, IL‐3, IL‐4, IL‐6, IL‐8, IL‐10, and IL‐12. The ABplex Multi‐Indicator Flow Co‐Analysis Service employs liquid microarray (flow fluorescence) technology by employing different color‐coded microspheres coupled to corresponding antibodies. The analysis was standardized and implemented by ABclonal Technologies, Inc. (Wuhan, China).

### 5.3 Enzyme‐Linked Immunosorbent Assay (ELISA)

BS and CSF from humans and rats, as well as the supernatants from C8D1A, primary microglia, and BV2 derived from mice, were collected and detected using ELISA kits. The analysis was performed according to the manufacturer's instructions. including IL‐3 human ELISA kit (EK0402, BOSTER, Wuhan, China), IL‐3 rat ELISA kit (EK1324, BOSTER, Wuhan, China), IL‐6 rat‐specific ELISA kit (EK0412, BOSTER, Wuhan, China), TNF‐α rat‐specific ELISA kit (EK0526, BOSTER, Wuhan, China), IL‐3 mouse ELISA kit (EK0403, BOSTER, Wuhan, China), IL‐6 mouse‐specific ELISA kit (EK0411, BOSTER, Wuhan, China), and TNF‐α mouse‐specific ELISA kit (EK0527, BOSTER, Wuhan, China).

### 5.4 Immunofluorescence Staining

Paraffin sections were prepared from rat brain tissue perfused with 4% paraformaldehyde. Brain tissue sections were deparaffinized in xylene and a series of graded ethanol solutions and then boiled in sodium citrate buffer (pH = 6) for 15 min to repair antigens. Brain tissue was perfused with PBS to prepare frozen sections. Mouse BV2 microglia and primary microglia were cultured on poly‐L‐lysine‐coated glass coverslips, then fixed with 4% paraformaldehyde. Rat brain tissue sections, frozen brain tissue sections, primary microglia, and mouse BV2 microglia cells were permeabilized with 0.3% Triton X‐100 solution (T8200, Solarbio) for 10 min and closed with normal sheep serum (Solarbio, Beijing, China) for 60 min. The primary and secondary antibody working solutions were prepared according to the instructions in the Antibody Handbook and incubated overnight at 4°C and for 60 min at room temperature. The primary and secondary antibodies include anti‐rabbit iNOS (18985‐1‐AP, Proteintech, 1:200), anti‐rabbit NRF2 (ab62352, Abcam, 1:400), anti‐rabbit P65 NF‐κB (8242S, CST, 1:400), and anti‐rabbit PRDX1 (15816‐1‐AP, Proteintech, 1:200), anti‐mouse IL‐3R (sc‐74522, santa, 1:50), anti‐mouse GFAP (3670, CST, 1:400), anti‐rabbit IL‐3 (PA5‐115414, Invitrogen, 1:400), anti‐mouse IBA1 (ab283319, abcam, 1:400), anti‐rabbit IL‐3R (GTX64388, GeneTex, 1:200), anti‐mouse MBP (66003‐1‐Ig, Proteintech, 1:500), anti‐mouse NEUN (MAB377, sigma, 1:1000), sheep anti‐mouse IgG H&L secondary antibody (Abcam, Alexa Fluor 488, ab 150113, 1:200), sheep anti‐mouse IgG H&L secondary antibody (Abcam, Alexa Fluor594, ab150116, 1:200), sheep anti‐rabbit IgG H&L secondary antibody (Abcam, Alexa Fluor 488, ab150077, 1:200), sheep anti‐rabbit IgG H&L secondary antibody (Abcam, Alexa Fluor 594, ab 150 080, 1:200), and Cell nuclei were stained with DAPI. Images were obtained using a Leica TCS SP8 confocal laser scanning microscope (Leica Microsystems, Wetzlar, Germany) and an electron microscope (Olympus, Tokyo, Japan). The average intensity of staining was determined by Image J software.ss.

### 5.5 Primary Microglia

Primary microglia were obtained from mouse mixed glial cultures using the shaking technique as previously described. The microglia were collected after 14 and 21 days of culture by agitating the flasks at 37°C for 2 h at a speed of 200 rpm. The isolated microglia were then re‐seeded at a density of (3 × 10^4^ /cm^2^) in DMEM/F12 (Corning, USA) containing 10% FBS. After allowing the cells to adhere for 48 h, Recombinant mouse IL‐ 3 protein (Active) (40 ng/ml) (Abcam) was added 6 h earlier than LPS (100 ng/ml) and IFN‐γ (25 ng/ml).

### 5.6 BV2 microglia, C8D1A Astrocytes, HEK293T culture

C8D1A cells were purchased from Zhongqiaoxinzhou Biotechnology (ZQXZ Biotechnology, Shanghai, China). BV2 microglia and HEK 293 T cells were purchased from Procell Life Science & Technology (Procell, Wuhan, China). The cell lines were cultured in Dulbecco's modified Eagle's medium (Gibco, CA, USA) supplemented with 10% fetal bovine serum (Gibco, CA, USA) and 1% penicillin‐streptomycin (Solarbio, Beijing, China) at 37 °C and 5% CO2. Then, BV2 microglia were transformed into pro‐inflammatory subtypes using LPS 100 ng/ml (MCE, Shanghai, China) and IFN‐γ 25 ng/ml (MCE, Shanghai, China). Recombinant mouse IL‐ 3 protein (Active) (40 ng/ml) (Abcam) was added 6 h earlier than LPS (100 ng/ml) and IFN‐γ (25 ng/ml). Astrocytes were induced into reactive glial cells using TNF‐α (MCE, Shanghai, China) (20 ng/ml) with IL‐1β (MCE, Shanghai, China) (20 ng/ml).

### 5.7 Animals and Surgery

All male rats (8–10 weeks old; weight 250 g) without specific pathogens were purchased from Jinan Pengyue Laboratory Animal Breeding Co., Ltd. (License No. SCXK [Lu] 2019 0003, Jinan, China). All animals were housed in a specific pathogen‐free animal house in the Central Hospital of Shandong First Medical University at a temperature of 23 ± 1°C, with a light/dark cycle of 12 h and free access to standard rat chow and sufficient water.

Animal operations were approved by the Laboratory Animal Welfare and Ethics Committee of the Affiliated Central Hospital of Shandong First Medical University on December 14, 2021 (approval number: JNCHIACUC2021‐75). All experiments were performed according to Animal Research: Reporting of In vivo Experiments (ARRIVE) guidelines [74].

### 5.8 To Establish a TBI Model

Rats were fasted for 12 h, anesthetized by intraperitoneal injection of 3% pentobarbital (30 mg/kg; Sigma‐Aldrich, USA), and fixed in a trauma model equipment frame. The head was dehaired and sterilized with povidone‐iodine. The scalp was incised to expose the left parietal bone, and brain tissue was exposed by a miniature cranial bone drill at 1.5 mm behind the coronal suture, and 2.5 mm to the left of the midline. A micro cranial drill (RWD, Shenzhen, China) was used to enlarge the bone window with a diameter of 5 mm, keeping the dura intact. The cranial impactor (RWD, 68 099, Shenzhen, China) was then used for striking according to the manufacturer's instructions; the parameters were: strike depth of 2 mm, speed of 3.5 m/s, and impact time of 300 m/s [75]. The incision was then sutured. The sham operation group only received the bone window opening without striking.

### 5.9 Stereotaxic Injection

Rats were anesthetized by intraperitoneal injection of 3% pentobarbital (30 mg/kg; Sigma‐Aldrich, USA). Recombinant Rat IL‐3 (carrier‐free) (Biolegend) was injected using a brain stereotaxic apparatus (RWD, Shenzhen, China) on the immediate and 3rd day of injury, respectively. The exact location of the localized cerebral cortex was 2.5 mm posterior and 2.5 mm lateral to the prefrontal lobe of the left hemisphere and 2 mm from the surface of the brain [76]. 5 µL Recombinant Rat IL‐3 was injected at 1 µL/min using a 10 µL Hamilton syringe (5 µg Recombinant Rat IL‐3 dissolved in 5 µL of normal saline at pH 7.2). The needle was left in place for an additional 5 min before being slowly withdrawn. The wound was then sutured, and the animal was allowed to regain consciousness.

### 5.10 Mechanical Allodynia

Paw withdrawal threshold in response to von Frey filament (Aesthesio; Danmic Global, USA) stimulation was measured to represent mechanical allodynia. After 3 consecutive days, the rats were placed in a Plexiglas chamber on a wire mesh floor and allowed to habituate for 10–15 min before the experiment. A series of thin wires was applied to the plantar median surface of the forelimb contralateral to the site of brain tissue injury in rats, and sustained pressure was applied to bending the wires for 5 s or to eliciting the paw withdrawal reflex for 5 s. Each thin wire was applied 5 times, and the 50% threshold (g) was calculated using the following formula: maximum bending force value − [(maximum bending force value − minimum bending force value)/ (positive rate of the maximum bending force − positive rate of the minimum bending force)] × (positive rate of the maximum bending force − 50%).

### 5.11 Inclined Plane Test

The rat is placed on a rectangular board held in place by rubber pads. The plate can be tilted, and the angle can be measured. The longitudinal axis of the rat's body was placed in parallel with the longitudinal axis of the inclined plate, and the rat's head was elevated to the side of the inclined plate. The angle of the inclined plate was changed by 5° each time, and the maximum angle of the inclined plate when the rat maintained the initial position for 5 s was taken as the measurement value. Three measurements were taken for each animal, and the average value was taken.

### 5.12 Forelimb Grip Strength Test

*The* grip force of the forepaws was measured using a grip force tester (Xin Xin, Shanghai, China). Grip force was used to determine the maximum peak force generated by a rat pulling out a metal rod. The machine was mounted on a stable and sturdy table, while the rat was allowed to grasp the metal rod with its front paws, while its tail was pulled backward in a horizontal plane. The peak tension, the force applied to the metal rod before it lost its grip, was recorded in grams. The rat was pulled three times, and the average of the three tests was recorded as the grip force [77].

### 5.13 Y‐Maze

Y‐maze testing was adapted from previously published protocols (39). The Y‐maze device consists of three arms connected in the center to form a Y shape. The walls of the arms were 10 cm high, and each side was labeled with a large black letter that served as a spatial landmark and clues. After closing one arm of the maze, rats were allowed to explore the other two arms for 5 min before being returned to their cages. After 20 min, rats were allowed to return to the Y‐shaped maze and explore all three arms over 5 min while being videotaped. The time spent on the new arm was quantified [78].

### 5.14 Modified Neurological Severity Score (mNSS)

The mNSS was performed as previously described [79]. The mNSS scores were administered to rats to assess neurological function on days (Bf‐T), day 7, day 14, day 21, day 28, and day 35. Neurological function, including motor and sensory systems, reflexes, and balance, was graded on a numerical scale from 0 to 18 (the higher the score, the more severe the neurological impairment, with a minimum mNSS score of 0 indicating no impairment).

### 5.15 T2‐Weighted Magnetic Resonance Imaging

Cranial MRI scan was performed on rats using a small animal 9.4‐T MRI scanner with a four‐channel surface coil (Bruker, 9.4T Biospec; Bruker BioSpin, Germany). Using an MR‐matched small animal anesthesia device, rats were anesthetized by inhalation using 1.5% isoflurane (RWD, Shenzhen, China) and then placed on a dedicated fixation system. The following parameters were used in the sequence protocol: T2‐weighted: TR: 2724 ms, TE:33 ms, number of slices: 26, slice thickness = 0.8 mm, FOV: 35 mm×35 mm, Matrix = 256 × 256, scan time: 5 min 48s 698 ms, T2‐weighted images in the sagittal plane were acquired using a Bruker ParaVision 6.0 system (Bruker, Ettlingen, Germany). Afterward, the mice were placed on a heating pad.

### 5.16 HE Stains

HE staining was performed using an HE staining kit (Servicebio, Wuhan, China), according to the manufacturer's instructions. Brain tissue sections were baked in an oven at 60°C for 1 h and then deparaffinized with xylene and a series of graded alcohol solutions. The sections are then stained with hematoxylin solution for 3 min and rinsed with tap water. After treatment with a hematoxylin differentiation solution, the sections were rinsed with tap water. The sections were then treated with hematoxylin Scott Tap Bluing reagent and rinsed with tap water. The sections were then dehydrated with 85% and 95% alcohol for 5 min and then stained with phenolphthalein. The sections were then stained with eosin dye solution for 5 min. Subsequently, the sections were dehydrated through a series of alcohol and xylene gradients and sealed with neutral gum. Images were observed and captured using an electron microscope (Olympus, Tokyo, Japan).

### 5.17 Nissl Staining

Nissl staining was performed according to the manufacturer's instructions (Beyotime, Shanghai, China). Brain tissue sections were deparaffinized with xylene and a series of graded ethanol solutions. The sections were stained with Nissl dye. Subsequently, the sections were dehydrated in a gradient series of alcohol solution and xylene and then sealed with neutral adhesive. Images were observed and captured using an electron microscope (Olympus, Tokyo, Japan).

### 5.18 Western Blotting (WB)

Cortical injury site brain tissue, primary microglia, and BV2 microglia were used to extract proteins using RIPA buffer (R0020, Solarbio) containing 1% (v/v) PMSF (P0100, Solarbio). Protein content was measured using the Enhanced BCA Protein Assay Kit (P0009, Beyotime Biotechnology). Cytoplasmic and nuclear proteins were extracted strictly according to the instructions of the Nuclear Protein Extraction Kit (R0050, Solarbio). Extracted proteins were separated by sodium dodecyl sulfate‐polyacrylamide gel electrophoresis (SDS‐PAGE) and then transferred to PVDF membranes (Millipore, Carrigtwohill, Ireland). Membranes were closed using non‐degreased milk and then incubated overnight at 4°C with a series of primary antibodies, including anti‐rabbit iNOS (18985‐1‐AP, Proteintech, 1:1000), anti‐rabbit‐NRF2 (ab62352, Abcam, 1:1000), anti‐rabbit phosphorylated P65 NF‐κB (3033s, CST, 1:1000), anti‐rabbit p65 NF‐κB (8242s, CST, 1:1000), anti‐rabbit IκB‐α (4812s, CST, 1:1000), anti‐rabbit Vinculin (26520‐1‐AP, Proteintech, 1:10000), anti‐rabbit LaminB1 (12987‐1‐AP, Proteintech, 1:10000), anti‐rabbit PRDX1 (15816‐1‐AP, Proteintech,1: 10000), anti‐mouse β‐actin (TA‐09, ZSGB‐BIO, 1:5000), anti‐rabbit KEAP1 (10503‐2‐AP, Proteintech 1:2000), anti‐rabbit HO‐1 (10701‐1‐AP, Proteintech, 1:3000), horseradish enzyme‐labelled goat anti‐rabbit IgG (ZB‐2301, ZSGB‐BIO, 1:5000), and horseradish enzyme‐labelled goat anti‐mouse IgG (ZB‐2305, ZSGB‐BIO, 1:5000). The primary fluorescent antibodies were the same as those used in immunofluorescence. The membrane was washed overnight and then incubated with the appropriate secondary antibody for 60 min at room temperature. Blots were detected by ECL chemiluminescent substrate (Biosharp, Hefei, China). Proteins were quantified by Image J software (National Institutes of Health, Bethesda, MD, USA).

### 5.19 Quantitative Real‐Time qPCR

AG RNAex Pro Reagent (Accurate Biology, Changsha, China) was used to extract total RNA from brain tissues and cells. By following the manufacturer's instructions, cDNA was synthesized with an Evo M‐MLV RT Mix Kit with gDNA Clean for qPCR (AG11728, ACCURATE BIOLOGY). The qPCR was performed with SYBR Green Premix Pro TagHS qPCR Kit (AG11701, ACCURATE BIOLOGY) and LightCycler 480 II (Roche, Indianapolis, Indiana, USA). Expression levels were normalized to an internal reference (GAPDH), and relative expression levels were calculated by the 2−ΔΔCT method [80]. All primer information is listed in Supporting Information.

### 5.20 RNA Sequencing and Bioinformatics Analyses

RNA sequencing (RNA‐seq) was performed by Biomarker Technologies (Beijing, China). The threshold for differentially expressed genes (DEG) was |logFC|>2 and adjusted p‐value < 0.05. Kyoto Encyclopedia of Genes and Genomes (KEGG) pathway analyses of the DEGs were performed using the Biomarker Technologies Cloud Platform. We thank BT Biotech, Ltd for assisting in sequencing and/or bioinformatics analysis. Using the BMKCloud tool at https://console.biocloud.net/, bioinformatics analyses were performed. The data that support the findings of this study are available from the corresponding author upon reasonable request.

### 5.21 GST Pull‐Down

BV2 cells were induced by IL‐3. GST‐tagged IL‐3R fusion proteins were purified using glutathione beads, and GST or GST‐IL‐3R proteins were added to the cell lysate. Cell lysates were transfected with 10 µg of Flag‐ARID 1A overnight at 4°C, followed by the addition of glutathione‐agarose 4 B beads for 3 h. After the removal of non‐specific binding proteins, the microbeads were eluted with a 2× SDS loading buffer. After overnight incubation at 4°C, the magnetic beads were washed four times with PBST, the proteins were eluted with the elution buffer, the beads were adsorbed on a magnetic rack, and the supernatant was collected. The recruited proteins were analyzed by WB stratified electrophoresis and Pulldown strip liquid chromatography mass spectrometry (LC‐MS/MS), and the recruited target proteins were screened.

### 5.22 Co‐Immunoprecipitation (CO‐IP)

Greater than 600 µg of protein lysate was collected for each sample. Lysates were preclarified using control agarose resin in the Pierce Co‐Precipitation Immunoprecipitation (Co‐IP) Kit (26 149, Thermo SCIENTIFIC). KEAP1 binding proteins were then captured with anti‐KEAP1 antibody (10503‐2‐AP, Proteintech 1:200) or protein IgG beads. NRF2 binding proteins were then captured with anti‐NRF2 antibody (66504‐1‐Ig, Proteintech 1:200) or protein IgG beads. IL‐3R binding protein was captured with anti‐IL‐3R antibody (sc‐74522, santa, 1:30) or protein IgG beads. PRDX1 binding protein was captured with anti‐PRDX1 antibody  or protein IgG beads(15816‐1‐AP, Proteintech,1: 50). Finally, the KEAP1‐NRF2 immune complex and IL‐3R‐PRDX1 complex were detected by WB analysis.

### 5.23 Stereotaxic Injections of AAV (Adeno‐Associated Virus Vectors)

Knockdown of AAV9‐IBA1 PRDX1 in rat cortex was purchased from Tsingke Biotechnology (Beijing, China). After anesthetizing the rats with isoflurane, the skin over the skull was incised, and a small hole was drilled in the skull above the target using a miniature skull drill (RWD, Shenzhen, China). AAVs were injected using a 10 µL Hamilton syringe, stereo tactically orientated to the cortex with the following coordinate system: (AP + 1.2 [locus 1], 0.3 [locus 2], ‐0.6 [locus 3]; ML + 5.5; DV – 3.5 mm from the skull) [81, 82]. The total amount of AAV solution was: cerebral cortex, 4.2 × 10^11^ vg/rat, speed 0.5 ul/min. To prevent reflux, the needle was left in place for a further 5 min, withdrawn for a short distance, and then left in the new position for a further 2 min before being removed. Transfection efficiency was verified after 21 days. Rats were allowed to recover for 3 weeks before TBI surgery.

### 5.24 Transfection of Plasmid and Lentiviruses

To investigate the functional implications of genetic manipulations in microglia, sgRNA was synthesized by RiboBio (Ruibiotech, Guangzhou, China). Lentiviral vectors were crafted to encompass both targeted gene knockout strategies (via PRDX1 Ctr, PRDX1 KO) (Ruibiotech, Guangzhou, China) for precise modulation of gene expression. These vectors were packaged in 293T cells, and the subsequent viral supernatants were purified and concentrated via ultracentrifugation, yielding high‐titer lentiviral particles. Prior to transduction, microglia were cultured to optimal density and maintained in a healthy state. Subsequently, the lentiviruses carrying these various constructs were individually introduced into microglia, enabling a comprehensive assessment of their functional consequences.

### 5.25 NRF2 Knockdown in Microglia

The microglia were cultured at a density of 1.0 × 10^6^ cells per dish in 100 mm Cell Culture Dishes for 12 h to cover 60% of the bottom area of each dish. Cells were pretreated with ML385 (10 µM) for 12 h. Cellular proteins were collected. NRF2 knockdown efficiency was monitored by WB.

### 5.26 Statistical Analysis

Each experiment was repeated with three or more biological proxies. Data are presented as mean ± standard deviation (mean ± standard deviation (SD). For comparisons involving more than two groups, one‐way analysis of variance (ANOVA) was performed. When the assumption of equal variances was met, Dunnett's t‐test was utilized for post‐hoc analysis; otherwise, the Kruskal‐Walli's method was applied. For comparisons between two groups, an independent t‐test was used. One‐way ANOVA and two‐way ANOVA, independent t‐test per‐P < 0.05 were used as the criterion for the significance of differences. Pearson correlation analysis was used to assess the correlation coefficients between IL‐3 and MRS, GCS. Using 3D Slicer to reconstruct MRI images and quantify the volume of lesion regions. The analysis was performed using GraphPad Prism (version: 9.4.0 (453), https://www.graphpad‐prism.cn/).

## Author Contributions

Designing research studies: B.N, Conducting experiments: N.N.H, Q.C.Z, Y.R.C, D.P.Y, J.N.K, X.F, Y.Z, S.L, C.Z,Y.P.F, Y.C.Y, Z.X.J. Acquiring data: N.N.H, Q.C.Z, Analyzing data: N.N.H, Q.C.Z, R.H.L, Y.F.J, Q.F.C, F.L, H.B, Y.X.Z, F,L. Providing reagents: Y.Z, Writing the manuscript: N.N.H, Q.C.Z.

## Conflicts of Interest

The authors declare no conflicts of interest.

## Supporting information




**Supporting File 1**: advs74772‐sup‐0001‐SuppMat.docx.

**Supporting File 2**: advs74772‐sup‐0002‐SuppInfo.pdf.

**Supporting File 3**: advs74772‐sup‐0003‐Data.xlsx.

## Data Availability

The data that support the findings of this study are available from the corresponding author upon reasonable request.;
