# Supplementary material for: IL‐3 Modulates Microglia Polarization and Attenuates Neuroinflammation in Traumatic Brain Injury
Source: Adv Sci (Weinh). 2026 Mar 31;13(29):e04511. doi: 10.1002/advs.202504511 (PMC13205870; doi:10.1002/advs.202504511)
Supplement: Supplementary file 1 — Supporting File 1: advs74772‐sup‐0001‐SuppMat.docx. [file ADVS-13-e04511-s002.docx]

**IL-3 Modulates Microglia Polarization and Attenuates Neuroinflammation in Traumatic Brain Injury**

*Nana Huang, Qingchen Zhang, Yanrui Chen, Dapeng Yu, Ronghan Liu, Jianning Kang, Xiang Fang, Ying Zhang, Hong Bian, Yanxin Zhao, Yongcheng Yin, Ce Zhang, Yanfei Jia, Qingfa Chen, Yuepeng Fang, Shang Li, Fang Li, Zhengxin Jin, Bin Ning^*^*

***^#^*** Co-first authors: *Nana Huang, Qingchen Zhang*

*** Corresponding author: *Bin Nin*

E-mail: bning@sdfmu.edu.cn (B. Ning).


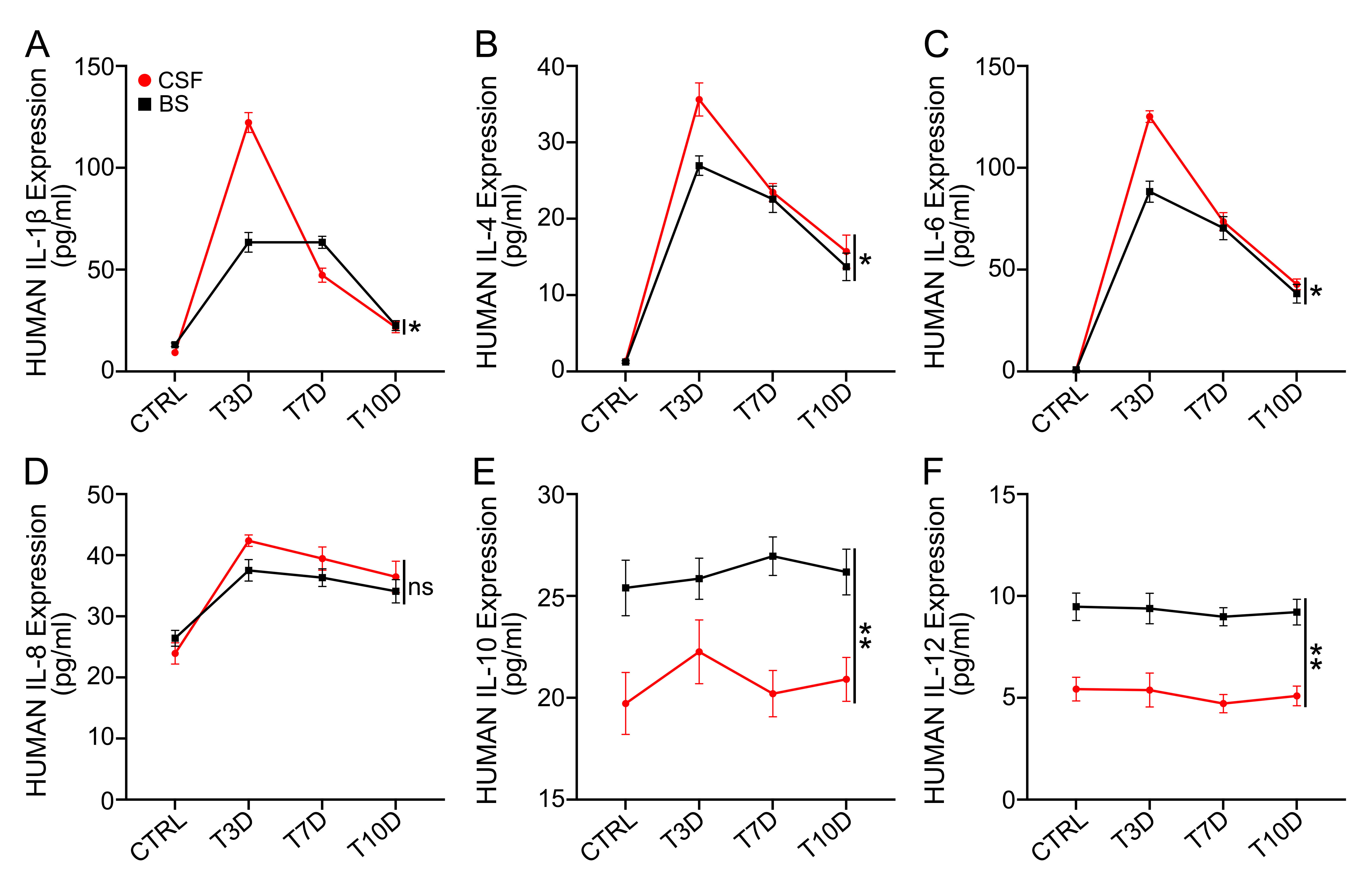


**Supplemental Figure S1.** Expression of inflammatory factors by ABplex multi-index flow. A-F) Differences in the expression of IL-1β, IL-4, IL-6, IL-8, IL-10, IL-12 in CSF and BS of TBI patients at different time points (day 3, day7, and day10 post-injury) compared to controls. *n*=20 per group*.* Summary data are presented as the mean ± SD (x ± s). ns, not statistically significant. **P*<0.05, ***P*<0.01, ****P*<0.001, *****P*<0.0001. BS: Blood serum, CSF: Cerebrospinal fluid.

**
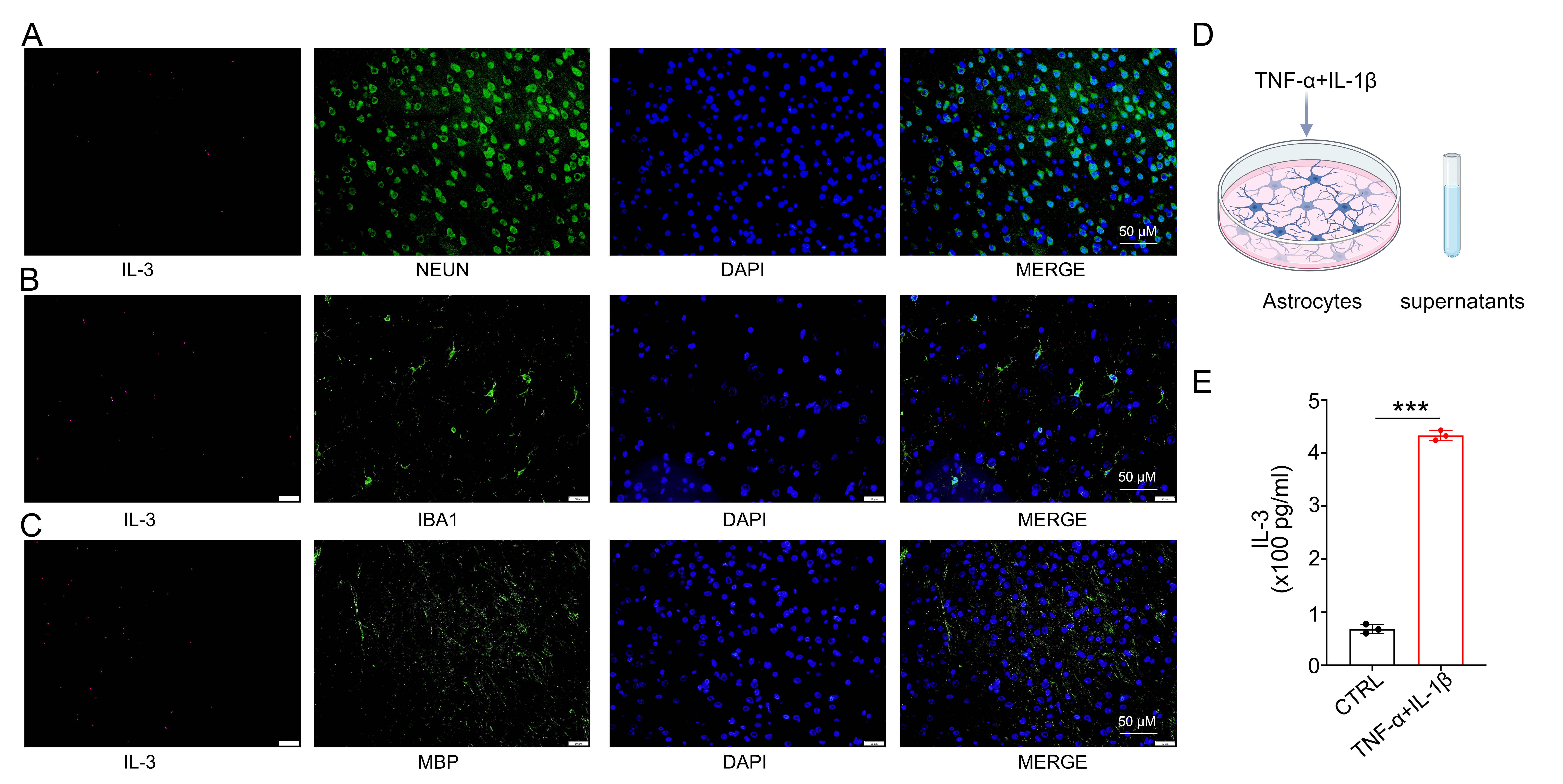
**

**Supplemental Figure S2.** IL-3 is predominantly secreted by the CNS and may be positively correlated with functional recovery in patients with TBI. A) Representative immunofluorescence (IF) images showing the expression levels of IL-3 and NeuN in TBI rat brains at day 7 post-injury. n=3 per group. B) Representative IF images showing the expression levels of IL-3 and IBA1 in TBI rat brains at day 7 post-injury. n=3 per group. C) Representative IF images showing the expression levels of IL-3 and MBP in TBI rat brains at day 7 post-injury. n=3 per group. D) Schematic Diagram of astrocytes Induction and Collection. E) The expression level of IL-3 in astrocytes treated with TNF-α (20 ng/ml) and Il-1β (20 ng/ml), detected by ELISA. *n*=3 per group*.* Summary data are presented as the mean ± SD (x ± s). ns, not statistically significant. **P*<0.05, ***P*<0.01, ****P*<0.001, *****P*<0.0001.


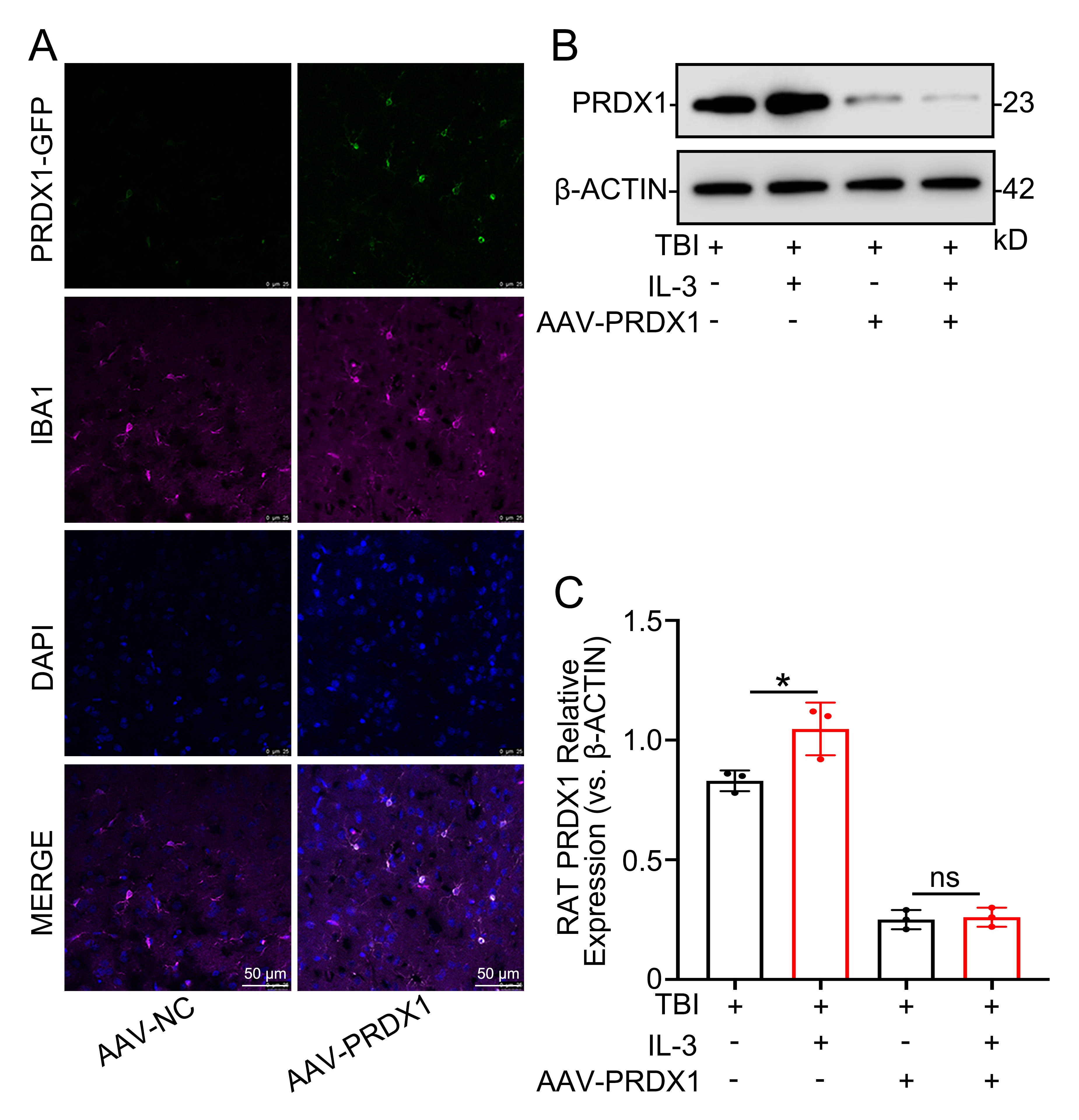


**Supplemental Figure S3.** *In vivo* PRDX1 knockdown efficiency. A) AAV transfection efficiency. B, C) Representative immunoblot images showing the expression levels of PRDX1 in the rat brain treated with or without IL-3 after AAV9-IBA1 PRDX1 injection. *n*=3 per group. Summary data are presented as the mean ± SD (x ± s). ns, not statistically significant. **P*<0.05, ***P*<0.01, ****P*<0.001, *****P*<0.0001. TBI: Traumatic brain injury. IL-3: Recombinant Rat IL-3 (20 μg/kg) was injected using a brain stereotaxic apparatus on the immediate and 3rd day of injury. AAV-NC: After transfecting with a negative control Adeno Associated Virus vector. AAV-PRDX1: After transfecting with a PRDX1-Knockdown Adeno Associated Virus vector. IL-3: Recombinant Rat IL-3 (20 μg/kg) was injected using a brain stereotaxic apparatus on the immediate and 3rd day of injury.

**
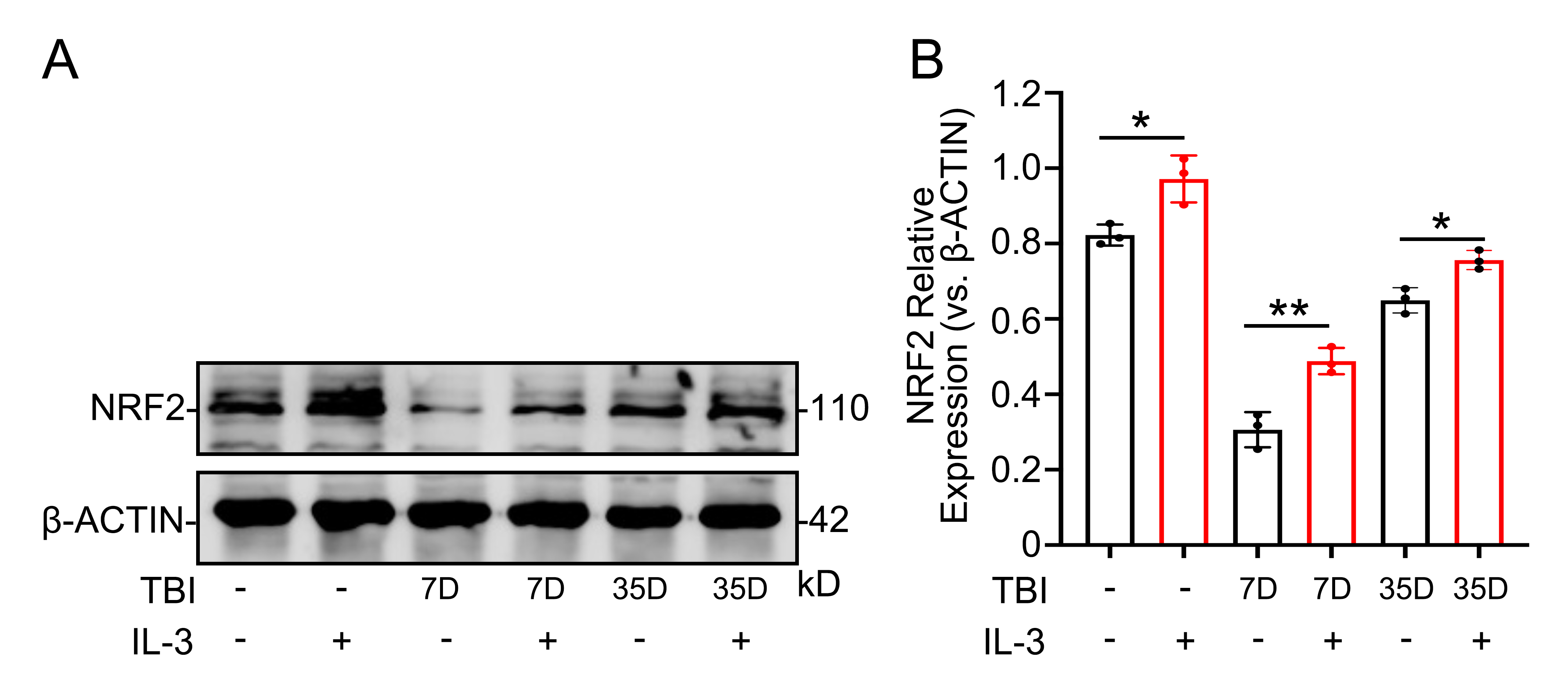
**

**Supplemental Figure S4.** The expression of NRF2 in *vivo* after IL-3 treatment. A, B) IL-3 upregulated the expression of NRF2 in rats with traumatic brain injury. *n*=3 per group. Summary data are presented as the mean ± SD (x ± s). ns, not statistically significant. **P*<0.05, ***P*<0.01, ****P*<0.001, *****P*<0.0001. TBI: Traumatic brain injury. IL-3: Recombinant Rat IL-3 (20 μg/kg) was injected using a brain stereotaxic apparatus on the immediate and 3rd day of injury.
