# Supplementary material for: IL‐3 Modulates Microglia Polarization and Attenuates Neuroinflammation in Traumatic Brain Injury
Source: Adv Sci (Weinh). 2026 Mar 31;13(29):e04511. doi: 10.1002/advs.202504511 (PMC13205870; doi:10.1002/advs.202504511)

Uncropped gels for Western Blots in Figure 2

F2-D

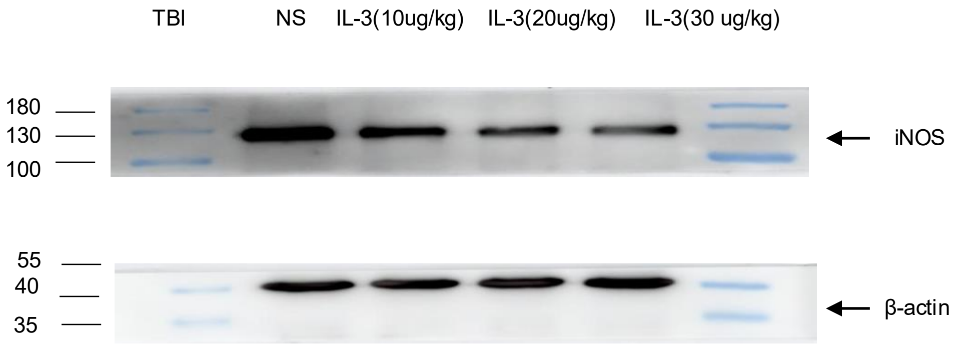

Uncropped gels for Western Blots in Figure 3

F3-C

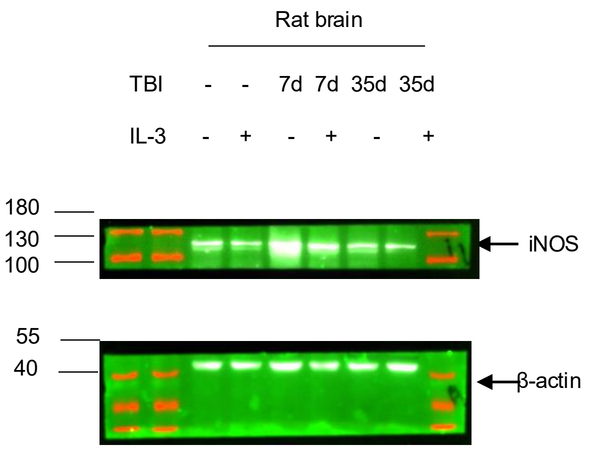

F3-H

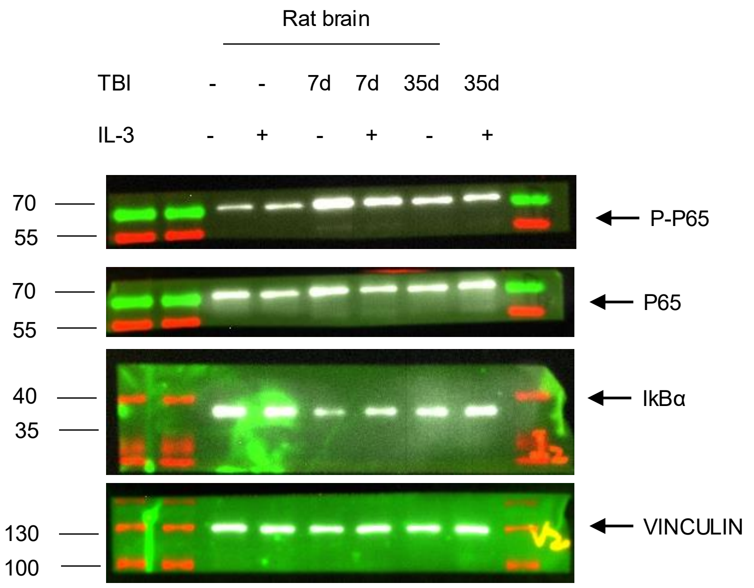

Uncropped gels for Western Blots in Figure 4

F4-C

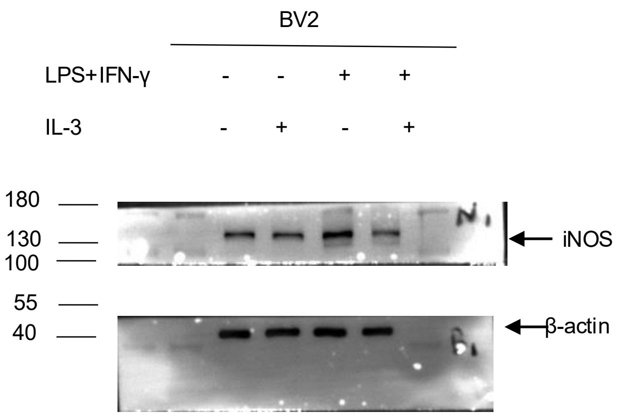

F4-M

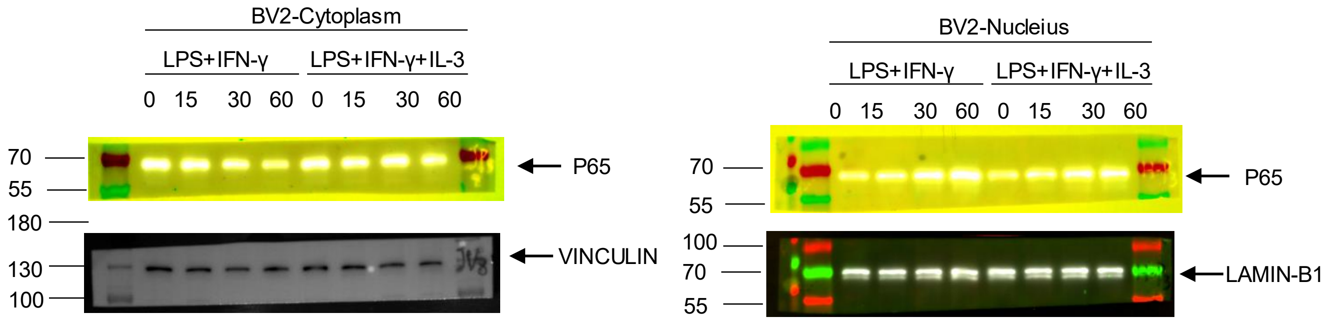

F4-P

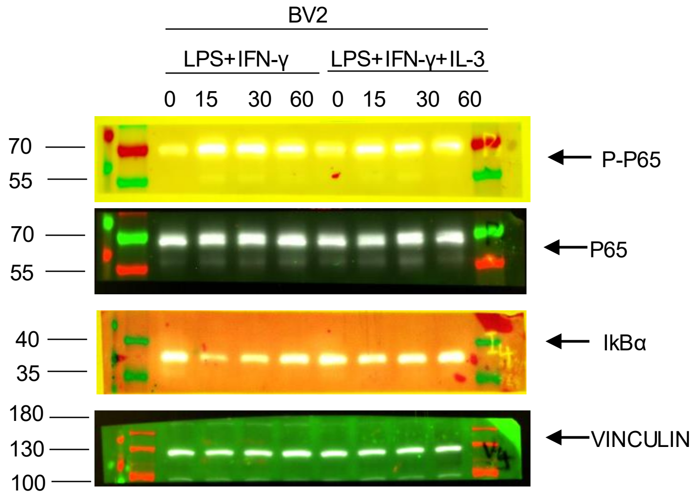

Uncropped gels for Western Blots in Figure 5

F5-D

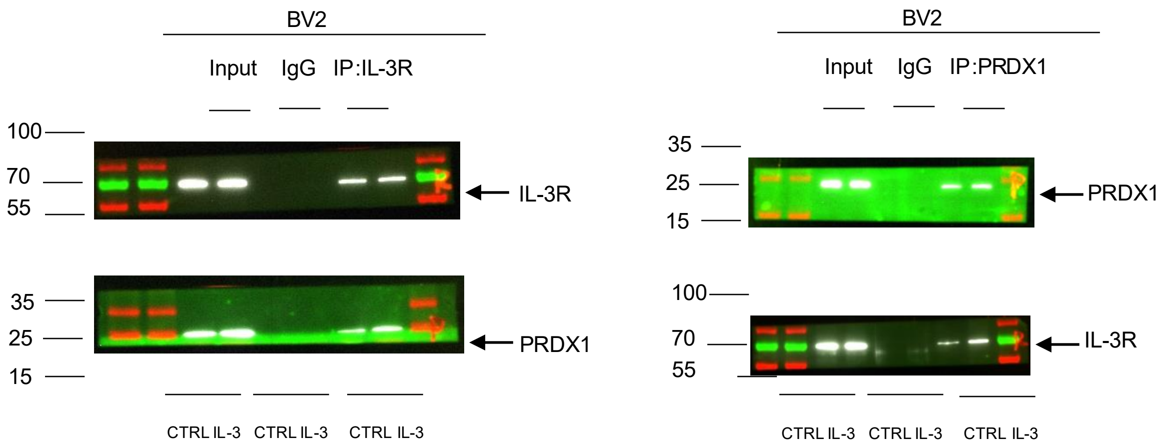

Uncropped gels for Western Blots in Figure 6

F6-O

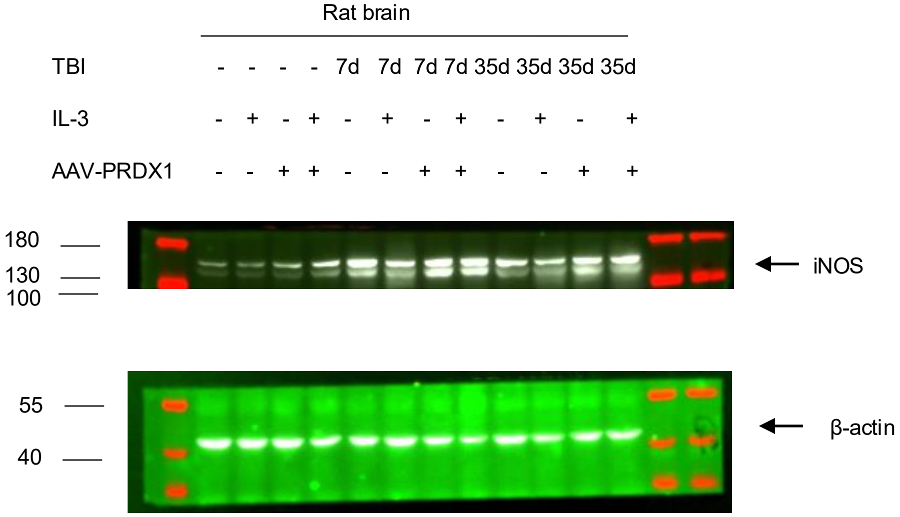

Uncropped gels for Western Blots in Figure 7

F7-B

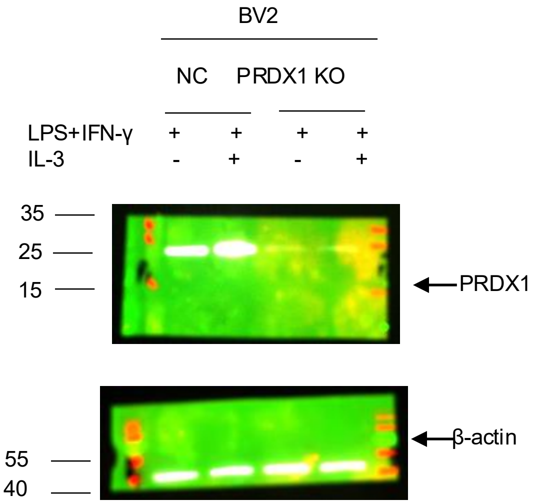

F7-F

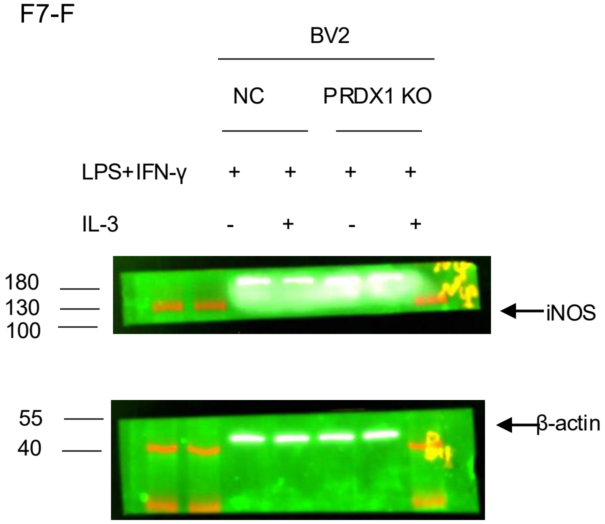

F7-L

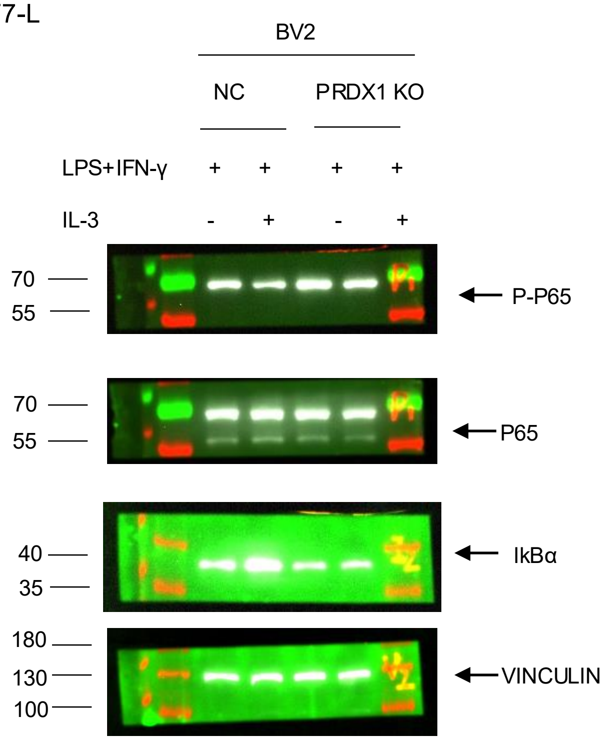

Uncropped gels for Western Blots in Figure 8

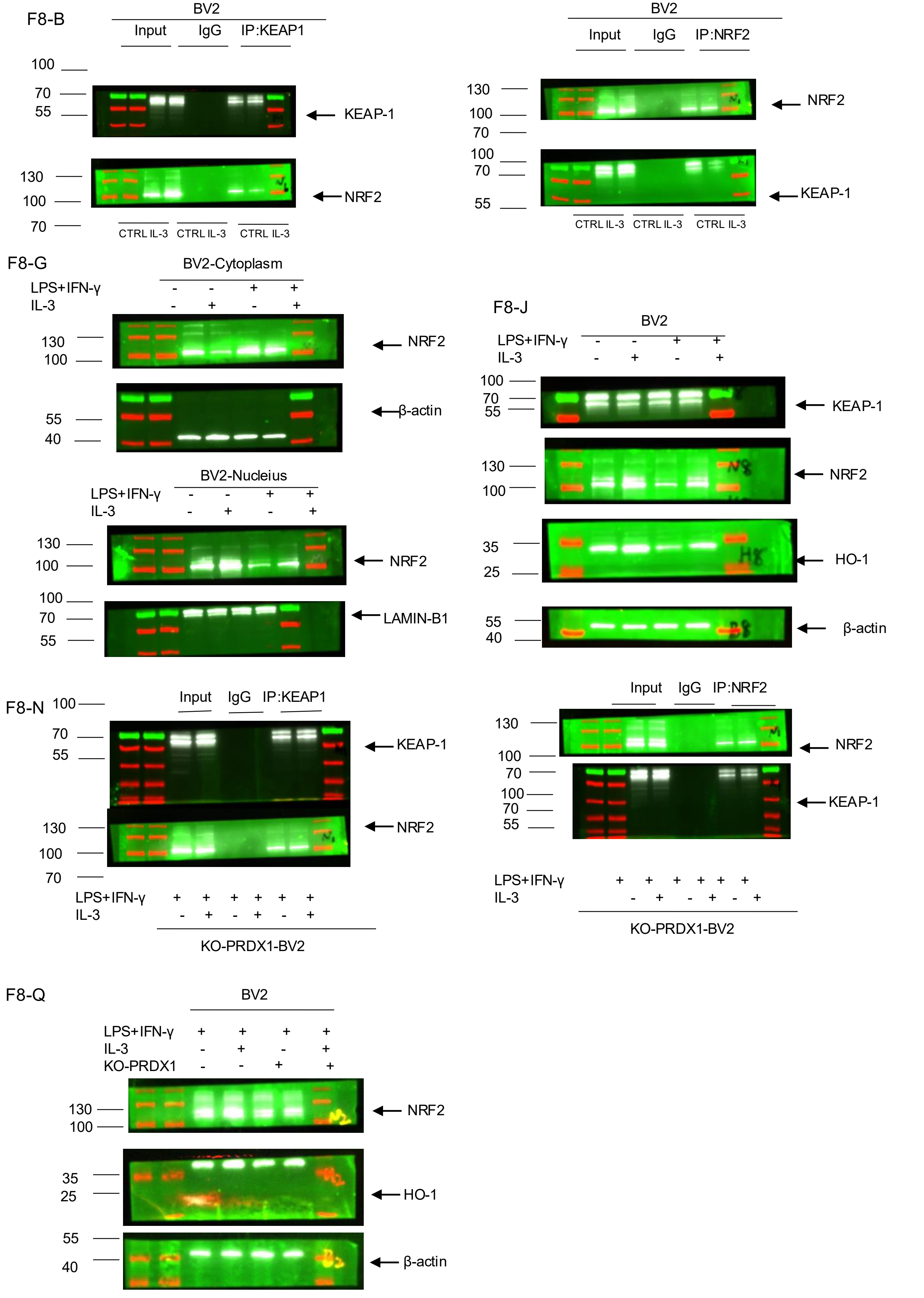

Uncropped gels for Western Blots in Figure 9

F9-B

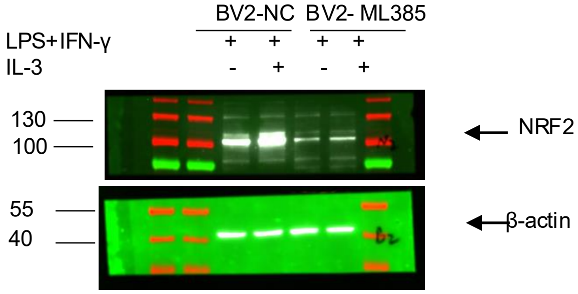

F9-F

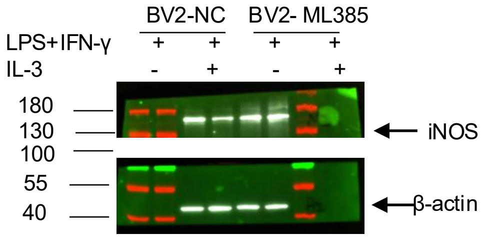

F9-L

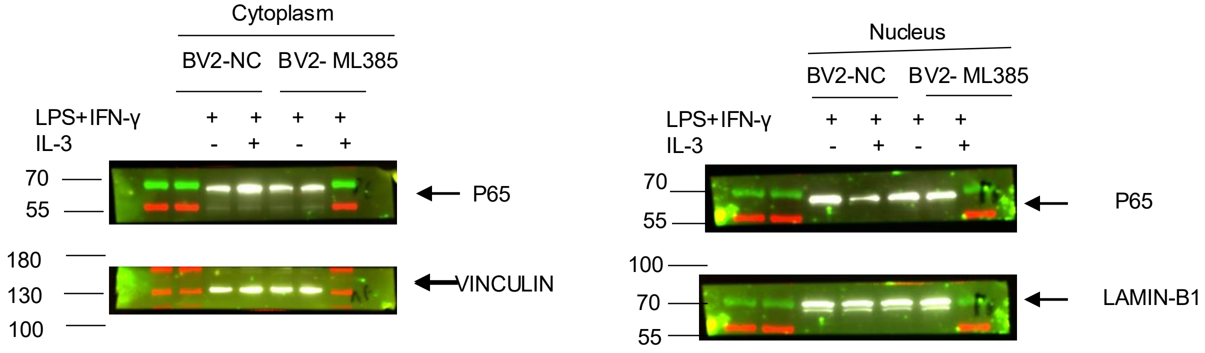

F9-O

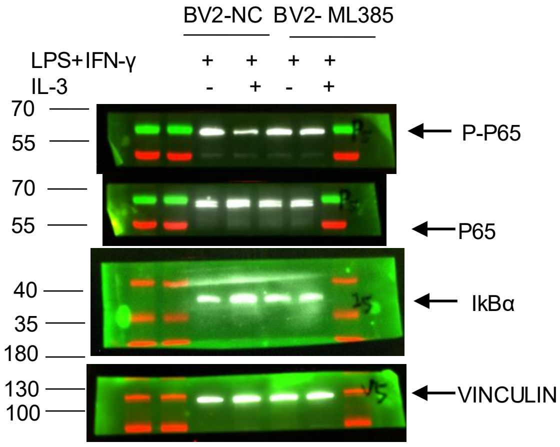

Uncropped gels for Western Blots in Supplemental Figure

Suppl.Figure 3-B

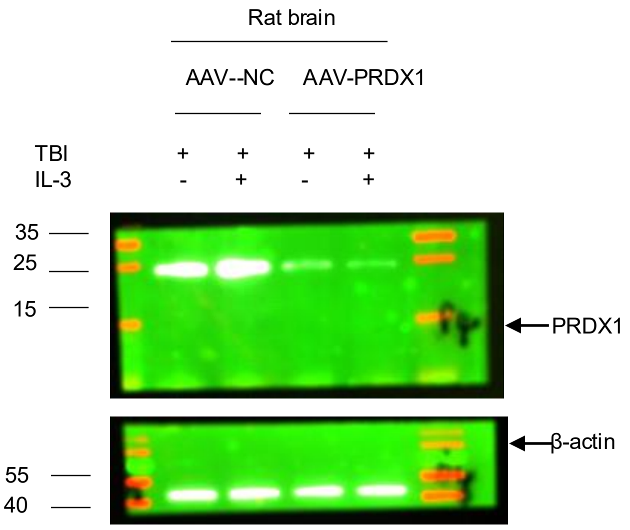

Uncropped gels for Western Blots in Supplemental Figure

Suppl.Figure 4-A

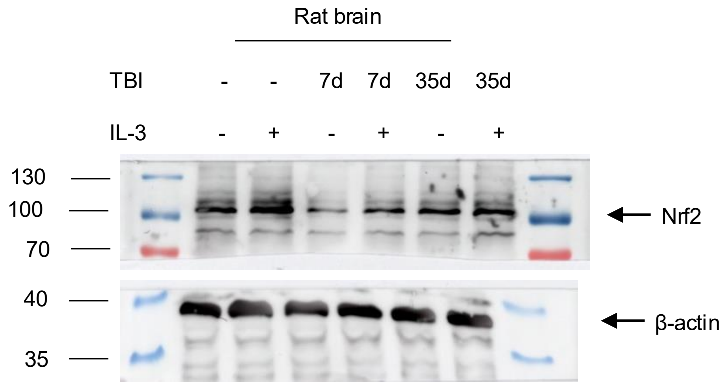

Supplement: Supplementary file 2 — Supporting File 2: advs74772‐sup‐0002‐SuppInfo.pdf. [file ADVS-13-e04511-s001.pdf]
